# Earthquake detection through computationally efficient similarity search

**DOI:** 10.1126/sciadv.1501057

**Published:** 2015-12-04

**Authors:** Clara E. Yoon, Ossian O’Reilly, Karianne J. Bergen, Gregory C. Beroza

**Affiliations:** 1 Department of Geophysics, Stanford University, Stanford, CA 94305, USA.; 2 Institute for Computational and Mathematical Engineering, Stanford University, Stanford, CA 94305, USA.

**Keywords:** computational seismology, earthquake detection, seismic monitoring, similarity search, locality-sensitive hashing, seismology, earthquakes

## Abstract

Seismology is experiencing rapid growth in the quantity of data, which has outpaced the development of processing algorithms. Earthquake detection—identification of seismic events in continuous data—is a fundamental operation for observational seismology. We developed an efficient method to detect earthquakes using waveform similarity that overcomes the disadvantages of existing detection methods. Our method, called Fingerprint And Similarity Thresholding (FAST), can analyze a week of continuous seismic waveform data in less than 2 hours, or 140 times faster than autocorrelation. FAST adapts a data mining algorithm, originally designed to identify similar audio clips within large databases; it first creates compact “fingerprints” of waveforms by extracting key discriminative features, then groups similar fingerprints together within a database to facilitate fast, scalable search for similar fingerprint pairs, and finally generates a list of earthquake detections. FAST detected most (21 of 24) cataloged earthquakes and 68 uncataloged earthquakes in 1 week of continuous data from a station located near the Calaveras Fault in central California, achieving detection performance comparable to that of autocorrelation, with some additional false detections. FAST is expected to realize its full potential when applied to extremely long duration data sets over a distributed network of seismic stations. The widespread application of FAST has the potential to aid in the discovery of unexpected seismic signals, improve seismic monitoring, and promote a greater understanding of a variety of earthquake processes.

## Teaser

Data mining techniques applied to continuous waveforms reveal many previously unknown small earthquakes.

## INTRODUCTION

Seismology, a data-driven science where breakthroughs often come from advances in observational capabilities (*1*), now has enormous data sets: years of continuous seismic data streams have been recorded on networks with up to thousands of sensors, and the rate of data acquisition continues to accelerate. Seismology can benefit from the development of new scalable algorithms that process and analyze these massive data volumes to extract as much useful information from them as possible. Our work focuses on improving earthquake detection using data mining techniques originally developed for audio recognition, image retrieval, and Web search engines.

### Background

A seismic network consists of multiple stations (receivers) at distributed locations, where each station has a seismometer continuously recording ground motion. Traditionally, an earthquake is detected at one station at a time, using an energy detector such as a short-term average (STA)/long-term average (LTA). STA/LTA computes the ratio of the STA energy in a short time window to the LTA energy in a longer time window, as these windows slide through the continuous data. A detection is declared when the STA/LTA ratio exceeds certain thresholds (*2*, *3*). An association algorithm then determines whether detections at multiple stations across the network are consistent with a seismic source. If a seismic event is detected at a minimum of four stations, it is included in an earthquake catalog, which is a database of the location, origin time, and magnitude of known earthquakes.

**Fig. 1:**
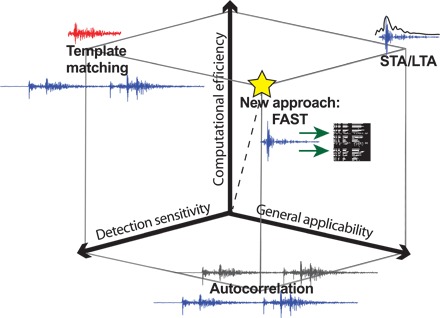
Comparison of earthquake detection methods in terms of three qualitative metrics: Detection sensitivity, general applicability, and computational efficiency. STA/LTA scores high on general applicability because it finds unknown sources, scores high on computational efficiency because it detects earthquakes in real time, but scores low on detection sensitivity because it can miss low-snr seismic events. Template matching rates high on detection sensitivity because cross-correlation can find low-snr events, rates high on computational efficiency because we only need to cross-correlate continuous data with a small set of template waveforms, but rates low on general applicability because template waveforms need to be determined in advance. Autocorrelation has high detection sensitivity because it cross-correlates waveforms, and high general applicability because it can find unknown similar sources, but has very low computational efficiency that scales poorly with the size of the continuous data set. FAST performs well with respect to all three metrics, combining the detection sensitivity and general applicability of correlation-based detection with high computational efficiency and scalability.

STA/LTA successfully identifies earthquakes with impulsive, high signal-to-noise ratio (snr) *P*-wave and *S*-wave arrivals. STA/LTA rates high on general applicability (Fig. 1), which we define as the ability to detect a wide variety of earthquakes without prior knowledge of the event waveform or source information. But STA/LTA fails to detect earthquakes, or may produce false detections, in more challenging situations such as low snr, waveforms with emergent arrivals, overlapping events, cultural noise, and sparse station spacing; thus, STA/LTA has low detection sensitivity (Fig. 1). Therefore, earthquake catalogs are incomplete for lower-magnitude events.

We can overcome the limitations of STA/LTA by taking advantage of information from the entire earthquake waveform for detection, rather than just the impulsive body wave arrivals. Seismic sources that repeat in time, over the course of weeks, months, or even years, have highly similar waveforms when recorded at the same station (*4*, *5*). Path effects are almost the same: searches for time-dependent travel time variations before (*6*) or after (*5*) large earthquakes reveal that temporal changes in Earth’s velocity structure are extremely subtle, so Earth’s structure is essentially constant at seismological time scales. Waveform cross-correlation exploits the resulting waveform similarity to perform as a sensitive earthquake detector.

Waveform cross-correlation, also called matched filtering or template matching, has proven to be a sensitive, discriminative method for finding a known seismic signal in noisy data; it scores high on detection sensitivity (Fig. 1). It is a “one-to-many” search method that computes the normalized correlation coefficient (CC) of a template waveform with successive candidate time windows of continuous waveform data, and any candidate window with a CC value above certain thresholds is considered a detection (*7*). The normalized CC between two time domain waveforms $\vec{a}$ and $\vec{b}$ is defined as

$$\text{CC}(\vec{a},\vec{b})=\frac{{\vec{a}}^{T}\vec{b}}{{\left\|\vec{a}\right\|}_{2}{\left\|\vec{b}\right\|}_{2}}=\frac{\sum_{i=1}^{M}a_{i}b_{i}}{\sqrt{\sum_{i=1}^{M}a_{i}a_{i}}\sqrt{\sum_{i=1}^{M}b_{i}b_{i}}}$$ (1)

where *M* is the number of samples in each waveform. Template matching allows detection of extremely low snr events, with few false positives, when the template includes waveforms from multiple channels and stations, and detection is based on the summed network CC (*7*, *8*). Template matching is a versatile and powerful technique that has found undetected events in a wide range of seismicity studies: uncataloged low-magnitude earthquakes (*9*), foreshocks (*10*), aftershocks (*11*), triggered earthquakes (*12*), earthquake swarms (*13*), low-frequency earthquakes (LFEs) in tectonic tremor (*8*) and triggered tremor (*14*), low-magnitude events in areas of potentially induced seismicity where seismic networks are sparse (*15*), nuclear monitoring and discrimination (*7*, *16*), and microseismic monitoring in geothermal (*17*) and oil and gas (*18*) reservoirs.

A major limitation of template matching, however, is that it requires an a priori waveform template; thus, it has low general applicability (Fig. 1). Templates are often chosen by extracting waveforms of catalog earthquakes or by picking out impulsive event waveforms from continuous data by human inspection. This is not an effective, comprehensive way to find unknown sources with low-snr repeating signals. The subspace detection (*19*) and empirical subspace detection (*20*) methods were developed to generalize template matching to similar, nonrepeating sources with more variation in their waveforms; however, we are interested in the most general case—systematically performing a blind search for signals with similar waveforms in continuous data without prior knowledge of the desired signal.

Autocorrelation is an exhaustive “many-to-many” search for similar waveforms when the desired signal waveform is unknown. We know that seismic signals of interest have a short duration (usually a few seconds on each channel), so we partition the continuous data into *N* short overlapping windows and cross-correlate all possible pairs of windows. Window pairs with CC exceeding a detection threshold are marked as candidate events, which can be postprocessed with additional cross-correlation, or grouped into “families” and stacked to form less noisy template waveforms. Autocorrelation has successfully found both known and previously unknown LFEs within tectonic tremor (*21*, *22*). Autocorrelation provides the improved sensitivity of waveform cross-correlation over STA/LTA and also enables detection of unknown sources with similar waveforms (Fig. 1).

Autocorrelation has a major disadvantage because it is computationally intensive (Fig. 1) and ultimately infeasible for detecting earthquakes in massive continuous data sets. For *N* windows, we must compute *N*(*N* − 1)/2 CCs to account for all possible window pairs; therefore the autocorrelation runtime scales quadratically with data duration, with algorithmic complexity *O*(*N*^2^). Autocorrelation performs a significant amount of redundant work because most pairs of windows are uncorrelated and not of interest for detection (fig. S1A); highly similar earthquakes detected by autocorrelation are a tiny fraction of the total number of pairs. Autocorrelation is well suited for detecting frequently repeating earthquakes in a few hours of continuous data (*21*), where *N* is small. But the *O*(*N*^2^) runtime of autocorrelation makes it impractical to find infrequently repeating events in days, weeks, months, or even years of continuous seismic data over a network of hundreds of channels and stations without using large-scale computing resources. We have developed a new approach that combines the strengths of autocorrelation (detection sensitivity and general ability to find unknown sources) and scalable runtimes for large *N* (Fig. 1). Our technique has the potential to improve earthquake monitoring and to reveal new insights into earthquake processes.

### New approach for earthquake detection

Many algorithms have been developed to efficiently search for similar items in massive data sets (*23*); applications include identifying similar files in a large file system (*24*), finding near-duplicate Web pages (*25*), detecting plagiarism in documents (*26*), and recognizing similar audio clips for music identification (*27*), such as in the Shazam mobile app (*28*). We can meet our objective of a fast, efficient, automated blind detection of similar earthquake waveforms in huge volumes of continuous data by leveraging scalable algorithms that are widely used in the computer science community. Seismologists are just beginning to exploit data-intensive search technologies to analyze seismograms; one recent application is an earthquake search engine for fast focal mechanism identification that retrieves a best-fit synthetic seismogram from a large database (*29*), whereas another study developed a fast-approximation algorithm to find similar event waveforms within large catalogs (*30*).

Locality-sensitive hashing (LSH), a widely used method for high-dimensional approximate nearest-neighbor search, allows us to avoid comparing dissimilar pairs, which constitute most pairs of waveforms in the data; LSH instead returns a shorter list of “candidate pairs” that are likely to be similar with high probability (*23*, *31*). In computer science, hashing is often used for the efficient insertion, search, and removal of items in databases, with constant *O*(*1*) runtime; each item is inserted into one hash bucket that is selected based on the output of a hash function (*32*). A hash table contains many hash buckets, and the hash function determines how items are distributed among the different hash buckets (*32*). With LSH (fig. S1B), we only need to search for pairs of similar items (seismic signals) within the same hash bucket—these pairs become candidate pairs, and we can ignore pairs of items that do not appear together in the same hash bucket, which comprise most pairs. Therefore, LSH allows search for similar items with a runtime that scales near-linearly with the number of windows from continuous data, which is much better than the quadratic scaling from autocorrelation.

Rather than directly comparing waveforms, we first perform feature extraction to condense each waveform into a compact “fingerprint” that retains only its key discriminative features. A fingerprint serves as a proxy for a waveform; thus, two similar waveforms should have similar fingerprints, and two dissimilar waveforms should have dissimilar fingerprints. We assign the fingerprints (rather than waveforms) to LSH hash buckets.

Our approach, an algorithm that we call Fingerprint And Similarity Thresholding (FAST), builds on the Waveprint audio fingerprinting algorithm (*33*), which combines computer-vision techniques and large-scale data processing methods to match similar audio clips. We modified the Waveprint algorithm based on the properties and requirements of our specific application of detecting similar earthquakes from continuous seismic data. We chose Waveprint for its demonstrated capabilities in audio identification and its ease of mapping the technology to our application. First, an audio signal resembles a seismogram in several ways: they are both continuous time series waveform data, and the signals of interest are often nonimpulsive. Second, Waveprint computes fingerprints using short overlapping audio clips, as in autocorrelation. Third, Waveprint takes advantage of LSH to search through only a small subset of fingerprints. Waveprint also reports fast retrieval results with high accuracy, and its feature extraction steps are easily parallelizable. FAST scores high on three qualitative desirable metrics for earthquake detection methods (Fig. 1) (detection sensitivity, general applicability, and computational efficiency), whereas other earthquake detection algorithms (STA/LTA, template matching, and autocorrelation) do well on only two of the three.

## RESULTS

### Data set

**Fig. 2:**
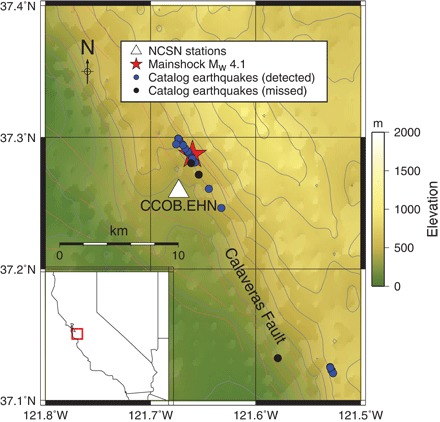
Map with locations of catalog earthquakes on the Calaveras Fault and seismic station with data. Double-difference catalog locations of the 8 January 2011 *M*_w_ 4.1 earthquake (red star) and NCSN catalog events (dots) between 8 and 15 January 2011 on the Calaveras Fault, and station CCOB.EHN (white triangle) from which we processed 1 week of data from 8 to 15 January 2011. Blue dots indicate the 21 catalog events detected by FAST, and black dots indicate the 3 catalog events missed by FAST. (**Inset**) Map location within California (red box).

We tested the detection capability of FAST on a continuous data set containing uncataloged earthquakes likely to have similar waveforms. The Calaveras Fault in central California (Fig. 2) is known to have repeating earthquakes (*34*). We retrieved 1 week (168 hours) of continuous waveform data, measured as velocity, from 8 January 2011 (00:00:00) to 15 January 2011 (00:00:00) at station CCOB.EHN (the horizontal north-south component) from the Northern California Seismic Network (NCSN). On 8 January 2011, a *M*_w_ 4.1 earthquake occurred on this fault, followed by several aftershocks according to the NCSN catalog. Most of these cataloged events were located within 3 km of the station.

We preprocessed the continuous time series data before running the FAST algorithm. We applied a 4- to 10-Hz bandpass filter to the data because correlated noise at lower frequencies interfered with our ability to detect uncataloged earthquakes. This correlated noise, which appears to be specific to the station, consists of similar nonseismic signals occurring at different times in the data. We then decimated the filtered data from their original sampling rate of 100 samples per second to 20 samples per second, so the Nyquist frequency is 10 Hz.

### FAST detection results

We demonstrate that FAST successfully detects uncataloged earthquakes in 1 week of continuous time series data, and we compare its detection performance and runtime against autocorrelation. Table 1 contains the parameters we used for FAST, and table S1 displays autocorrelation parameters; although these parameters are not tuned to their optimal values, they work reasonably well. Generally, we do not expect event times from FAST, autocorrelation, and the catalog, which each have their own lists of event detection times, to match exactly. Therefore, for comparison purposes, we define matching events as occurring within 19 s of each other (Table 1), which is the maximum time of overlap between a 10-s-long fingerprint with a 1-s lag (Table 1) and a 10-s-long autocorrelation window (table S1).

**Table 1:** FAST input parameters. These were used for detection in synthetic data (except the event detection threshold) and in 1 week of CCOB.EHN data.

**FAST parameter**	**Value**
Time series window length for spectrogram generation	200 samples (10 s)
Time series window lag for spectrogram generation	2 samples (0.1 s)
Spectral image window length	100 samples (10 s)
Spectral image window lag = fingerprint sampling period	10 samples (1 s)
Number of top *k* amplitude standardized Haar coefficients	800
LSH: number of hash functions per hash table *r*	5
LSH: number of hash tables *b*	100
Initial pair threshold: number *v* (fraction) of tables, pair in same bucket	4 (4/100 = 0.04)
Event detection threshold: number *v* (fraction) of tables, pair in same bucket	19 (19/100 = 0.19)
Similarity search: near-repeat exclusion parameter	5 samples (5 s)
Near-duplicate pair and event elimination time window	21 s
Autocorrelation and catalog comparison time window	19 s

**Fig. 3:**
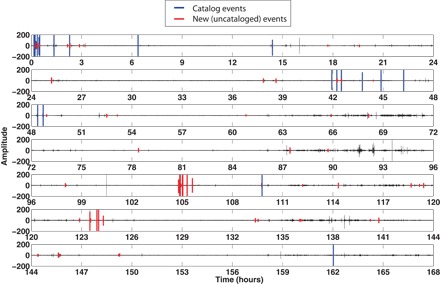
FAST event detections plotted on 1 week of continuous data. Data are from station CCOB.EHN (bandpass, 4 to 10 Hz) starting on 8 January 2011 (00:00:00). FAST detected a total of 89 earthquakes, including 21 of 24 catalog events (blue) and 68 new events (red).

Table 2 summarizes the performance of autocorrelation and FAST in terms of several metrics: number of detected events, false detections, catalog detections, new (uncataloged) detections, missed detections, and runtime. FAST detected a total of 89 earthquakes in these data (Fig. 3), whereas autocorrelation found 86 events; thus, they have comparable performance in terms of the total number of detected events. FAST has more false detections than autocorrelation, but runs much faster. Most events are detected by both autocorrelation (64 of 86) and FAST (64 of 89), but a considerable fraction of new events are found by either autocorrelation (22 events) or FAST (25 events) but not by both.

**Table 2:** Summary of performance comparison between autocorrelation and FAST for several metrics. The numbers for metrics 3 to 5 should sum to the number in metric 1.

**Metric**	**Autocorrelation**	**FAST**
1. Total number of detected events	86	89
2. Number of false detections (false positives)	0	12
3. Number and percentage of catalog detections	24/24 = 100%	21/24 = 87.5%
4. Number of new detections from both algorithms	43	43
5. Number of new detections from one, missed by the other	19	25
6. Number of missed detections (false negatives)	25	22
7. Runtime	9 days 13 hours	1 hour 36 min

FAST detected 21 of 24 catalog events (Fig. 3) located within the region of interest in Fig. 2 (between 37.1° and 37.4°N and between 121.8° and 121.5°W), whereas autocorrelation found all 24. Neither autocorrelation nor FAST detected catalog events outside this region, using data from only CCOB.EHN. Figure S2 shows 20-s normalized waveforms ordered by catalog event time for the 21 catalog events found by FAST (fig. S2A), with magnitudes ranging from *M*_w_ 4.10 for the mainshock to *M*_d_ 0.84 for the smallest event (table S2), and for the 3 catalog events missed by FAST, which are false negatives (fig. S2B). FAST did not detect these three catalog events because they did not repeat within the week of continuous data (Fig. 2). One event at 361,736 s was found at a location (37.13208°N and −121.57879°W) different from the other catalog events. The other two events at 314,077 and 336,727 s were located closer to most of the catalog events near the mainshock but had shallower depths (3.50 and 3.53 km, respectively) compared to most of the catalog events with depths of 6 to 7 km (table S2). Autocorrelation found these three catalog events because their initial phase arrival matched that of another earthquake with high CC; however, inspection of the earthquake pair after 5 s revealed that the rest of their waveforms were dissimilar (fig. S3), so it is not surprising that FAST did not detect them.

In addition to the 21 catalog events, FAST also detected 68 new events that were not in the catalog (Fig. 3). These additional events provide a more complete description of seismicity on the Calaveras Fault; the higher temporal resolution of this aftershock sequence can potentially be used to more reliably predict aftershock rates for epidemic-type aftershock sequence models. Figure S4 shows 20-s normalized waveforms from these new events ordered by event detection time in 1 week of CCOB.EHN data. FAST detected 43 new events that autocorrelation also found (fig. S4A), as well as 25 new events that autocorrelation missed (fig. S4B). These events are noisier than the catalog event waveforms in fig. S2.

The waveforms in fig. S4 are not properly aligned in time for two reasons: first, FAST event times are accurate only up to 1 s, equal to the time lag between adjacent fingerprints (Table 1), and second, there can be multiple detection times for the same event, and we consider only the time with the highest FAST similarity (Supplementary Materials). FAST similarity is defined as the fraction of hash tables with the fingerprint pair in the same bucket (Materials and Methods). FAST does not estimate a precise arrival time, but this can easily be computed with cross-correlation in a subsequent step in the detection pipeline.

We also estimated the number of false-positive and false-negative detections made by FAST, given our choice of parameters in Table 1. The estimation was based on a careful visual inspection of waveforms: waveforms had to look like an impulsive earthquake signal on all three components of data at station CCOB to be classified as “true detections,” although FAST used only the EHN channel for detection. In our application, we wanted to only detect earthquakes, so we did not classify similar signals having nonimpulsive waveforms as true detections. FAST returned 12 false-positive detections above the event detection threshold that were visually identified as low-amplitude noise from their 20-s normalized waveforms (fig. S5A). Autocorrelation did not have any false positives because we deliberately set a high detection threshold (CC = 0.818); we could have set a lower detection threshold for autocorrelation to detect more events, but this would also introduce false positives that complicate the automated comparison between FAST and autocorrelation detections. FAST failed to detect 19 uncataloged events (fig. S5B) found by autocorrelation, so these are false negatives. Ten of these 19 detections were missed for the same reason as the three catalog events (fig. S3): autocorrelation matched the initial *P*-wave arrivals, but the entire waveforms were dissimilar. FAST missed a total of 22 events (including the three catalog events) that autocorrelation found. But the 25 new events found by FAST and missed by autocorrelation can be interpreted as false negatives for autocorrelation; their CC values ranged from 0.672 to 0.807, so they were below the CC = 0.818 threshold. The overall shapes of the waveform pairs for these 25 events are similar but not precisely aligned in time (fig. S6).

Finally, we compare the serial runtime performance of FAST against autocorrelation to detect events in 1 week of CCOB.EHN data. Autocorrelation took 9 days and 13 hours to produce a list of earthquake detections, whereas FAST took only 1 hour and 36 min, a 143-fold speedup when processed on an Intel Xeon Processor E5-2620 (2.1-GHz central processing unit). The speedup factor estimate has some uncertainty because neither autocorrelation nor FAST implementations were optimized for the fastest possible runtime. FAST spent 38% of its time in feature extraction, 11% in database generation, and 51% in similarity search. FAST has an enormous advantage over autocorrelation in terms of runtime, and based on the scalability of these two algorithms, we expect this advantage to increase for longer-duration continuous data sets.

Figure S7 illustrates the small number of candidate pairs output from FAST, which contributes to its computational efficiency. It displays a histogram of similar fingerprint pairs (including near-duplicate pairs) on a log scale, binned by FAST similarity. There are *N*_fp_(*N*_fp_ − 1)/2 ~ 1.8 × 10^11^ possible fingerprint pairs, but FAST outputs 978,904 pairs with a similarity of at least the initial threshold of 0.04 (Table 1), which constitute only 0.0005% of the total number of pairs. After applying the event detection similarity threshold of 0.19 (Table 1), we retain only 918 pairs. Further postprocessing (Supplementary Materials) returns a list of 101 detections that includes 89 true events and 12 false detections: removing near-duplicate pairs reduced the number of pairs to 105, and removing near-duplicate events reduced the number of detections from 2 × 105 = 210 to 101. Although FAST incurs some runtime overhead by computing fingerprints with feature extraction, it is small compared to the speedup achieved from avoiding unnecessary comparisons.

## DISCUSSION

### Scaling to large data sets

To quantify the scalability of FAST runtime and memory usage for larger data sets, we downloaded 6 months (181 days) of continuous data from station CCOB.EHN (from 1 January 2011 to 30 June 2011) and ran FAST on seven different data durations ranging from 1 day to 6 months (table S3), including 1 week. For this scaling test, we used the parameters in Table 1, but we increased the number of hash functions *r* from 5 to 7. This parameter change decreases the detection performance but improves the computational efficiency.

**Fig. 4:**
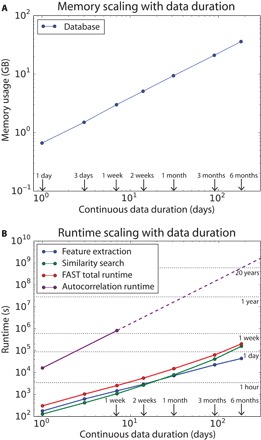
FAST scaling properties as a function of continuous data duration up to 6 months. (**A**) Memory usage for the database generated by LSH. (**B**) FAST total runtime (red) subdivided into runtime for feature extraction (blue) and similarity search (green). Autocorrelation runtimes (purple) for continuous data longer than 1 week are extrapolated based on quadratic scaling (dashed line). These results are from running FAST with the parameters in Table 1, with the number of hash functions *r* increased from 5 to 7, which decreased the total runtime for 1 week of continuous data to under an hour.

The memory usage of the LSH-generated database depends on the number of hash tables, the number of fingerprints, and additional overhead specific to the hash table implementation. We used the Linux top command to estimate the memory usage for long-duration data. We found that about 36 GB of memory was required for 6 months of continuous data (Fig. 4A).

We investigated FAST runtime as a function of continuous data duration by separately measuring the wall clock time for the feature extraction and similarity search steps (Fig. 4B). Feature extraction scales linearly with data duration, whereas similarity search scales near-linearly as *O*(*N*^1.36^). For comparison, we recorded autocorrelation runtime for up to 1 week of data, then extrapolated to longer durations by assuming quadratic scaling. FAST can detect similar earthquakes within 6 months of continuous data in only 2 days and 8.5 hours—at least three orders of magnitude faster than our autocorrelation implementation, which is expected to require about 20 years to accomplish the same task.

### Limitations

FAST trades off higher memory requirements in exchange for faster runtime and reduced algorithmic complexity. Unlike autocorrelation, FAST needs a significant amount of memory because the LSH-generated database stores hash tables, with each hash table containing references to all *N*_fp_ = 604,781 fingerprints that are distributed among its collection of hash buckets. These memory requirements increase as we anticipate searching for events in months to years of continuous data. For years of continuous data, memory may become a bottleneck, and a parallel implementation of the database in a distributed computing environment would be necessary.

We can improve the detection sensitivity and thresholding algorithm for FAST in several ways. Our current implementation used an event detection threshold of 0.19 (Table 1) for the FAST similarity metric, which was set by visually inspecting waveforms: most events above this threshold looked like earthquakes, and most events below it looked like noise. As we process longer-duration continuous data, we will require an automated and adaptive detection threshold that varies with the noise level during a particular time period. We do not want to compromise detection sensitivity for an entire year of continuous data by using an elevated constant detection threshold because of a short, unusually noisy time period. Also, the similar fingerprint pairs output from FAST (fig. S8) are really candidate pairs (*23*) that require additional postprocessing to be classified as event detections. For example, instead of using the FAST similarity event detection threshold of 0.19 (Table 1), we can take all pairs above the initial threshold 0.04 (Table 1) and set an event detection threshold based on directly computed CC for waveforms of candidate pairs.

Similar fingerprint pairs output from FAST (fig. S8) only identify pairwise similarity between waveforms; however, we would like to find groups of three or more waveforms that are similar to each other. Clustered similarity has useful seismological applications, from identifying repeating earthquake sequences to finding LFE families in tectonic tremor, which can include thousands of similar events during an episode (*8*, *21*, *22*). Future postprocessing steps could be developed to determine “links” between pairs of similar waveforms to create groups of similar waveforms. Further research into identifying connections between pairs that repeat multiple times (*22*), or applying a combination of clustering and graph algorithms (*30*) often used to analyze social networks (*23*), could help solve this problem.

FAST is designed to find similar signals in continuous data, but these signals may not necessarily be due to earthquakes. FAST may also register detections for correlated noise, especially if the data have repeating noise signals such as the 12 false detections in fig. S5A. We applied a 4- to 10-Hz bandpass filter to the CCOB.EHN data because low-frequency correlated noise, specific to this station, degraded our detection performance. Other examples of correlated noise include short-duration, high-amplitude glitches, spikes, and other artifacts. Correlated noise also negatively impacts autocorrelation event detection: when we did not apply the 4- to 10-Hz bandpass filter to the continuous data, autocorrelation also detected many nonseismic signals with higher similarity than the earthquake waveforms. Possible strategies to mitigate the effect of correlated noise include the following: applying an adaptive detection threshold that is higher during noisy periods, grouping detections in a way that separates similar seismic signals from similar noise signals, and developing postprocessing algorithms such as feature classifiers (*35*) that discriminate earthquakes from noise.

Because FAST is designed to detect similar signals, we do not expect it to find a distinct earthquake signal that does not resemble any other signals in the continuous data processed by FAST. For example, if the data contain 100 event signals but only two of them have similar waveforms, FAST would return only two detections. Longer-duration continuous data are more likely to contain similar earthquake signals, so FAST would be able to detect more seismic events. If the data still contain a distinct, nonrepeating earthquake signal, STA/LTA can be used to detect it, provided that it has an impulsive arrival with enough energy. In addition, FAST can be applied in “template matching mode,” a variant not pursued in this study, in which fingerprints from a section of continuous data are queried against a database of fingerprints from template signals extracted from other data sets, enabling detections similar to known waveforms without requiring the matching signal to appear during the continuous data interval.

### Conclusions and future implications

Seismology is a data-driven science where new advances in understanding often result from observations (*1*) and the amount of data collected by seismic networks has never been greater than today. Computer scientists have pioneered data mining algorithms for similarity search, with applications ranging from audio clips, to images in large databases, to Internet Web pages. FAST demonstrates that we can harness these algorithms to address a fundamental problem in seismology: identifying unknown earthquakes.

The most important advantage of FAST over competing approaches is its fast runtime and scalability. For 1 week of continuous data, FAST runs about 140 times faster than autocorrelation while detecting about the same total number of events. For longer continuous data streams, however, we anticipate that serial FAST would run orders of magnitude faster than autocorrelation, based purely on the runtime complexity of these algorithms: quadratic for autocorrelation and near-linear for FAST (Fig. 4B).

Seismologists have previously applied parallel processing to speed up template matching on graphics processing units (*36*) and on a Hadoop cluster (*37*). We also use a parallel autocorrelation implementation (Supplementary Materials) as a reference for comparing FAST detection results. FAST runtime can be further reduced with a parallel implementation, although only the feature extraction steps are embarrassingly parallel; distributing the LSH-generated fingerprint database across multiple nodes requires a nontrivial algorithm redesign.

To be able to detect earthquakes in low-snr environments, FAST needs to be applied to distributed seismic networks. The existing FAST algorithm detected events from one channel of continuous data at a single station (CCOB); we are developing an extension of FAST that can detect events using all three components from one station and can incorporate multiple stations. Many template matching studies (*7*, *8*, *11*) have demonstrated that incorporating channels from multiple stations enhances detection sensitivity, revealing low-snr signals buried in noise. In addition, a coherent signal recorded at multiple stations, at different distances and azimuths from the source, is more likely to be an earthquake rather than noise local to the station. Therefore, we expect a multiple-station detection method to reduce the number of false detections and to mitigate the negative effect of correlated noise on FAST detection performance, assuming that correlated noise in time is independent between different stations. Any detection method needs to be robust to changes in network architecture, such as the addition of new stations or station dropouts for long-duration data.

The detection capability of FAST needs to be explored further through tests on a variety of data sets that pose challenges for detection as a result of low snr, waveforms with nonimpulsive arrivals, overlapping waveforms, and correlated noise. Future work also ought to develop more discriminative fingerprinting and to explore different ways to hash fingerprints into the database.

Because FAST can identify similar seismic events given a query event in near-constant time, the technique may also be applicable to real-time earthquake monitoring. The increased detection sensitivity could reduce catalog completeness magnitudes if implemented at a large scale across a seismic network. A real-time FAST implementation could store a database of fingerprints from the continuous data, and as new data stream in, new fingerprints would be created, added to the database, checked for similarity with other fingerprints, and classified as a detection or not. FAST can also enable large-scale template matching: hundreds of thousands of template fingerprints can be used as search queries to a massive database of fingerprints. FAST may find similar earthquakes missed by STA/LTA or template matching in a diverse range of earthquake sequences: foreshocks, aftershocks, triggered earthquakes, swarms, LFEs, volcanic activity, and induced seismicity. FAST could also identify low-magnitude seismic signals that repeat infrequently, perhaps once every few months.

## MATERIALS AND METHODS

The FAST algorithm detects similar signals within a single channel of continuous seismic time series data. It has two major components: (i) feature extraction and (ii) similarity search. Feature extraction compresses the time series data by converting each waveform into a sparse, binary fingerprint. All of the fingerprints are inserted into a database using locality-sensitive hash functions. Given a desired “search query” fingerprint, the database returns the most similar matching fingerprints with high probability in near-constant time (*23*). In our current many-to-many search application, we use every fingerprint in the database as a search query so that we can find all pairs of similar fingerprints within the data set; however, we can also select a subset of fingerprints or use other sources of data as search queries. Finally, the most dissimilar pairs returned from the search queries are removed, and additional postprocessing and thresholding (Supplementary Materials) result in a list of earthquake detection times.

### Fingerprinting: Feature extraction

**Fig. 5:**
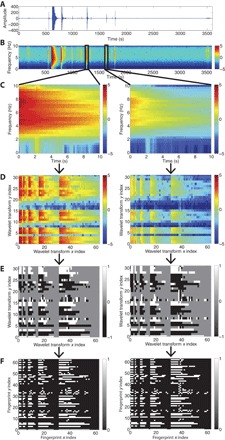
Feature extraction steps in FAST. (**A**) Continuous time series data. (**B**) Spectrogram: amplitude on log scale. (**C**) Spectral images from two similar earthquakes at 1267 and 1629 s. (**D**) Haar wavelet coefficients: amplitude on log scale. (**E**) Sign of top standardized Haar coefficients after data compression. (**F**) Binary fingerprint: output of feature extraction. Notice that similar spectral images result in similar fingerprints.

Figure 5 (A to F) contains an overview of feature extraction steps in FAST, which follows most of the workflow in Baluja *et al.* (*33*): continuous time series data (A), spectrogram (B), spectral image (C), Haar wavelet transform (D), data compression (E), and binary fingerprint (F).

#### Spectrogram

We compute the spectrogram (Fig. 5B) of the time series data (Fig. 5A) using the short-time Fourier transform (STFT). We take overlapping 10-s windows in the time series (separated by a 0.1-s time lag) (Table 1), apply a Hamming tapering function to each window, and compute the Fourier transform of each tapered window. We calculate the power (squared amplitude) of the resulting complex STFT, then downsample the spectrogram to 32 frequency bins, which smooth away some noise. Earthquakes appear in the spectrogram as transient, high-energy events (Fig. 5B).

#### Spectral image

We want to compare and detect similar earthquakes, which are short-duration signals, so we divide the spectrogram into overlapping windows in the time dimension and refer to each window as a “spectral image.” Matching patterns between spectral images has been previously proposed as an earthquake detection method (*38*). The spectral image of an earthquake signal has high power (Fig. 5C) compared to the rest of the spectrogram (Fig. 5B). There are also window length and lag parameters for spectral images; we chose *L*_fp_ = 100 samples for the spectral image length and τ_fp_ = 10 samples for the lag between adjacent spectral images (Table 1), which correspond to a spectral image length of 10 s and a spectral image lag of 1 s. A shorter spectral image lag increases detection sensitivity and timing precision at the expense of additional runtime. The total number of spectral image windows, and ultimately the number of fingerprints *N*_fp_, is

$$N_{\text{fp}}=[\frac{N_{t}-(L_{\text{fp}}-\tau_{\text{fp}})}{\tau_{\text{fp}}}]$$ (2)

where *N*_t_ is the number of time samples in the spectrogram. For the week of continuous data from CCOB, *N*_fp_ = 604,781.

Because the spectrogram content varies slowly with time (*33*), we can find similar seismic signals with a longer spectral image lag of 1 s, compared to the 0.1-s lag used in time series autocorrelation, which contributes to the fast runtime of FAST. We have fewer spectral images (compared to the number of autocorrelation time windows) from the same duration of continuous data; thus we have fewer fingerprints to first calculate and then compare for similarity.

Although the spectral image length is 10 s, it includes 20 s of waveform data. Each of the *L*_fp_ = 100 time samples in the spectral image contains 10 s of data, with an offset of 0.1 s between each sample.

The next step (Haar wavelet transform) requires each spectral image dimension to be a power of 2. We therefore downsample in the time dimension from *L*_fp_ = 100 to 2^6^ = 64 samples. We previously downsampled in the frequency dimension to 2^5^ = 32 samples, so the final dimensions of each spectral image are 32 samples by 64 samples.

#### Haar wavelet transform

We next compute the two-dimensional Haar wavelet transform of each spectral image to get its wavelet representation, which facilitates lossy image data compression with a fast algorithm while remaining robust to small noise perturbations (*33*, *39*). Figure 5D displays the amplitude of the Haar wavelet coefficients of the spectral images from Fig. 5C; an earthquake signal has high power in the wavelet coefficients at all resolutions, appearing in a distinct pattern.

Wavelets are a mathematical tool for multiresolution analysis: they hierarchically decompose data into their overall average shape and successive levels of detail describing deviations from the average shape, from the coarsest to the finest resolution (*40*). In Fig. 5D, the finest resolution detail coefficients are in the upper-right quadrant, and they get coarser as we move diagonally left and down, until we reach the average coefficient of the entire spectral image in the lower-left corner. A Fourier transform has basis functions that are sines and cosines, is localized only in the frequency domain, and can describe periodic signals using just a few coefficients. In an analogous way, a discrete wavelet transform (DWT) has different kinds of basis functions, is localized in both the time and the frequency domains, and can express nonstationary, burst-like signals (such as earthquakes) using only a few wavelet coefficients (*41*). The DWT has previously been used to improve STA/LTA earthquake detection and to accurately estimate phase arrivals (*42*). The DWT can be computed recursively using the fast wavelet transform but requires the dimension of the input data to be a power of 2. The DWT can also be computed with other wavelet basis functions, such as the Daubechies basis functions of different orders (*41*), but this requires more computational effort than the Haar basis.

#### Data compression: Wavelet coefficient selection

We now compress the data by selecting a small fraction of the Haar wavelet coefficients for each spectral image, discarding the rest. Because much of the continuous signal is noise, we expect diagnostic wavelet coefficients for earthquakes to deviate from those of noise. Therefore, we keep the top *k* Haar wavelet coefficients with the highest deviation from their average values, with deviation quantified by standardizing each Haar coefficient. We use *z*-score–based standardized coefficients, rather than simple amplitudes of the Haar coefficients, because they have greater discriminative value and empirically resulted in improved earthquake detection performance.

We now describe how standardized Haar coefficients can be obtained. The *M* = 32 × 64 = 2048 Haar coefficients of *N* = *N*_fp_ spectral images are placed in a matrix *H* in *ℜ*^*M*×*N*^. Let *Ĥ* be the matrix with columns *ĥ*_*j*_ = *h*_*j*_/‖*h*_*j*_‖_2_, obtained by normalizing each column *h*_*j*_ of *H*. Then, for each row *i* of the matrix, we compute the sample mean μ_*i*_ and corrected sample standard deviation σ_*i*_ for Haar coefficient *i* over all spectral images *j*

$$\mu_{i}=\frac{1}{N}(\sum_{j=1}^{N}{\hat{H}}_{ij}),\sigma_{i}=\sqrt{\frac{1}{N-1}(\sum_{j=1}^{N}{\left({\hat{H}}_{ij}-\mu_{i}\right)}^{2})}$$ (3)

The standardized Haar coefficient *Z*_*ij*_, computed as the *z*-score for each Haar coefficient *i* and spectral image *j*, then gives the number of standard deviations from the mean across the data set for that coefficient value

$${\hat{Z}}_{ij}=\frac{{\hat{H}}_{ij}-\mu_{i}}{\sigma_{i}}$$ (4)

For each spectral image, we select only the top *k* = 800 standardized Haar coefficients (Table 1, 800/2048 = 39%) with the largest amplitude (which preserves negative *z*-scores with a large amplitude) and set the rest of the coefficients to 0. Only the sign of the top *k* coefficients is retained (Fig. 5E): +1 for positive (white), −1 for negative (black), and 0 for discarded coefficients (gray). Storing the sign instead of the amplitude provides additional data compression while remaining robust to noise degradation (*33*, *39*).

#### Binary fingerprint

We generate a fingerprint that is binary (consists of only 0 and 1) and sparse (mostly 0) so that we can use the LSH algorithm described in the next section to efficiently search for similar fingerprints and to minimize the number of bits required for storage. We represent the sign of each standardized Haar coefficient using 2 bits: −1 → 01, 0 → 00, 1 → 10. Thus, each fingerprint uses twice as many bits as Haar coefficients. Because each spectral image window had 2048 Haar coefficients, each fingerprint has 2 × 2048 = 4096 bits. Figure 5F shows the binary fingerprints derived from the earthquake spectral images, where 1 is white and 0 is black.

### Similarity search

After feature extraction, we have a collection of *N*_fp_ fingerprints, one for each spectral image (and thus the waveform). Our objective is to identify pairs of similar fingerprints to detect earthquakes. FAST first generates a database in which similar fingerprints are grouped together into the same hash bucket with high probability. Then, in similarity search, the database returns all fingerprints that are similar to a given search query fingerprint, as measured by Jaccard similarity. The search is fast and scalable with increasing database size, with near-constant runtime for a single search query. FAST uses every fingerprint in the database as a search query, so the total runtime is near-linear.

#### Jaccard similarity

In template matching and autocorrelation, we use the normalized CC (Eq. 1) to measure the similarity between two time domain waveforms. Here, we use the Jaccard similarity as a similarity metric for comparing fingerprints in the LSH implementation. The Jaccard similarity of two binary fingerprints *A* and *B* is defined as (*23*)

$$J(A,B)=\frac{\left|A\cap B\right|}{\left|A\cup B\right|}$$ (5)

In Eq. 5, the numerator contains the number of bits in both *A* and *B* that are equal to 1, whereas the denominator is the number of bits in either *A*, *B*, or both *A* and *B* that are equal to 1. Figure S9A shows two very similar normalized earthquake waveforms, and fig. S9B displays the Jaccard similarity of their corresponding fingerprints.

#### Database generation

The dimensionality of each fingerprint is reduced from a 4096-element bit vector to a shorter integer array using an algorithm called min-wise independent permutation (Min-Hash) (*43*). Min-Hash uses multiple random hash functions *h*_*i*_(*x*) with permutation *i*, where each hash function maps any sparse, binary, high-dimensional fingerprint *x* to one integer *h*_*i*_(*x*). Min-Hash has an important LSH property—the probability of two fingerprints *A* and *B* mapping to the same integer is equal to their Jaccard similarity

Pr[h(A)=h(B)]=J(A,B) (6)

Thus, Min-Hash reduces dimensionality while preserving the similarity between *A* and *B* in a probabilistic manner (*23*, *33*).

**Fig. 6:**
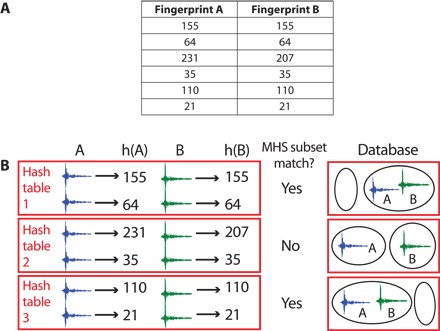
Example of how LSH groups fingerprints together in the database. (**A**) Example of MHS for two similar fingerprints *A* and *B*, with *p* = 6. (**B**) LSH decides how to place two similar fingerprints *A* (blue) and *B* (green) into hash buckets (ovals) in each hash table (red boxes); waveforms are shown for easy visualization. The MHS length is *p* = 6, and there are *b* = 3 hash tables, so each hash table gets a different subset of the MHS of each fingerprint that is 6/3 = 2 integers long: the output of *r* = 2 Min-Hash functions. Taking each hash table separately: if the MHS subsets of *A* and *B* are equal, then *A* and *B* enter the same hash bucket in the database; this is true in hash tables 1 and 3, where *h*(*A*) = *h*(*B*) = [155, 64] and *h*(*A*) = *h*(*B*) = [110, 21], respectively. In hash table 2, however, the MHS subsets of *A* and *B* are not equal, because *h*(*A*) = [231, 35] and *h*(*B*) = [207, 35], so *A* and *B* enter different hash buckets.

The output of Min-Hash is an array of *p* unsigned integers called a Min-Hash signature (MHS), given a sparse binary fingerprint as input (*23*). The MHS can be used to estimate the Jaccard similarity between fingerprints *A* and *B* by counting the number of matching integers from the MHS of both *A* and *B*, then dividing by *p*; the Jaccard similarity estimate improves as *p* increases (*33*). Each of the *p* integers is computed using a different random hash function *h*_*i*_ applied to the same fingerprint. These *p* Min-Hash functions are constructed by drawing *p* × 4096 (where 4096 is the number of bits in the fingerprint) independent and identically distributed random samples from a uniform distribution, returned by calling a uniform random hash function, to get an array *r*(*i*,*j*), where *i* = 1,…,*p* and *j* = 1,…,4096. Then, to obtain the output of a Min-Hash function *h*_*i*_(*x*) for a given fingerprint *x*, we use the index of the *k* nonzero bits in the fingerprint to select *k* values in the *r*(*i*,*j*) array. For example, if we consider the first hash function *h*_1_(*x*) out of all *p* hash functions and if the index of a nonzero bit in fingerprint *x* is *j* = 4, then *r*(1, 4) is chosen. Out of all the *k* selected values *r*(*i*,*j*), we select the minimum value and assign the index *j* that obtains the minimum as the output of the Min-Hash function *h*_*i*_(*x*) (*23*). We further reduce the output size by keeping only 8 bits so that the MHS has a total of 8*p* bits; each integer in the MHS has a value between 0 and 255 (*33*). Figure 6A shows sample MHS arrays for two similar fingerprints *A* and *B*, with *p* = 6.

LSH uses the MHS to insert each fingerprint into the database. Figure 6B demonstrates how LSH places two similar fingerprints *A* and *B* into hash buckets in each hash table in the database, given their MHS arrays (Fig. 6A). The 8*p* bits of each MHS are partitioned into *b* subsets with 8*r* bits in each subset (*p* = *rb*). These 8*r* bits are concatenated to generate a hash key, which belongs to exactly one out of *b* hash tables. Each hash key is a 64-bit integer index that retrieves a hash bucket, which can contain multiple values (references to fingerprints). For a given hash table, if *A* and *B* share the same hash key (equivalently, if their MHS subsets in Fig. 6 are the same), then they are inserted into the same hash bucket; otherwise, they are inserted into different hash buckets (*23*, *33*).

**Fig. 7:**
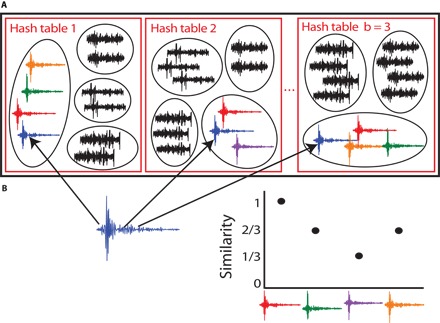
LSH database and similarity search example. (**A**) Database generated using LSH, with *b* = 3 hash tables (red boxes); each hash table has many hash buckets (ovals). LSH groups similar fingerprints into the same hash bucket with high probability; earthquake signals (colors) are likely to enter the same bucket, whereas noise (black) is grouped into different buckets. (**B**) Search for waveforms in database similar to query waveform (blue). First, LSH determines which bucket in each hash table has a waveform that matches the query. Next, we take all other waveforms in the same bucket in each hash table and calculate the FAST similarity between each (query, database) waveform pair: the fraction of hash tables containing the pair in the same bucket. The red waveform is in the same bucket as the blue query waveform in all three hash tables, so their similarity is 1; the green waveform is in the same bucket in two of three hash tables; and so on. This figure displays waveforms for easy visualization, but the database stores references to fingerprints in the hash buckets, and a search query requires converting the waveform to its fingerprint.

Figure 7A presents a schematic of a database created by LSH; highly similar fingerprints are likely to be grouped together in the same hash bucket. The database stores 32-bit integer references to fingerprints in the hash buckets, rather than the fingerprints (or waveforms) themselves. We generate *bN*_fp_ hash keys and values for each MHS from all *N*_fp_ fingerprints and insert all of the values into hash buckets within the *b* hash tables, given their corresponding hash keys. Each of the *N*_fp_ = 604,781 fingerprints is represented in some hash bucket in every hash table, so LSH produces multiple groupings of fingerprints into hash buckets.

#### Similarity search within the database

The LSH-generated database provides a fast, efficient way to find similar fingerprints and, therefore, similar waveforms. Figure 7B shows how one can search for fingerprints in the database that are similar to the query fingerprint (blue). For each search query fingerprint, we determine its hash bucket in each hash table using the procedure illustrated in Fig. 6 and retrieve all fingerprint references contained in these selected hash buckets using the corresponding hash keys, forming pairs where the first item is the query fingerprint and the second item is a similar fingerprint from the same hash bucket in the database (*23*, *33*). Thus, for each query, we perform as many lookups as hash tables. The amortized time for a lookup is *O*(1). The retrieval time depends on the number of items per bucket; it is desirable for each bucket to contain a small subset of fingerprint references in the database, so that we ignore fingerprint references in all other hash buckets, making the search scalable with increasing database size. Out of all retrieved pairs for a given query, we only retain the pairs that appear in at least *v* = 4 out of *b* = 100 hash tables, for an initial FAST similarity threshold of 4/100 = 0.04 (Table 1); these become our candidate pairs (*33*). We later set *v* = 19 out of *b* = 100 hash tables, for a FAST similarity of 0.19, as an event detection threshold (Table 1) after visual inspection of waveforms corresponding to these fingerprint pairs. We define FAST similarity as the fraction of hash tables containing the fingerprint pair in the same hash bucket.

The theoretical probability of a successful search—the probability that two fingerprints hash to the same bucket (have a hash collision) in at least *v* out of *b* hash tables, with *r* hash functions per table, as a function of their Jaccard similarity *s*—is given by (*23*)

$$\text{Pr}\;=1-\sum_{i=0}^{v-1}[(\begin{array}{c} b\\ i \end{array}){\left(1-s^{r}\right)}^{b-i}{\left(s^{r}\right)}^{i}]$$ (7)

$$(\begin{array}{c} b\\ i \end{array})=\frac{b!}{i!(b-i)!}$$ (8)

The red curves in fig. S10 (same in all subplots) plot Eq. 7 with varying Jaccard similarity *s*, given our specific input parameters (Table 1): *r* = 5 hash functions per table, *b* = 100 hash tables (so the MHS for each fingerprint had *p* = *rb* = 500 integers), and *v* = 19 as the threshold for the number of hash tables containing a fingerprint pair in the same bucket. The probability increases monotonically with similarity.

We adjust the position and slope of the curve from Eq. 7 by varying the *r*, *b*, and *v* parameters so we can modify the Jaccard similarity at which we have a 50% probability of a successful search. Figure S10A modifies *r* while keeping *b* and *v* constant; the curve shifts to the right as *r* increases, requiring higher Jaccard similarity for a successful search. For a large *r*, there are a large number of hash buckets; thus, the resulting low density of fingerprints within these buckets may lead to missed detections, as similar fingerprints are more likely to end up in different buckets. But if *r* is too small, there are few hash buckets; each bucket may have too many fingerprints, which would increase both the runtime to search for similar fingerprints and the likelihood of false detections. Figure S10B modifies *b*, keeping *r* and *v* constant; the curve moves left as *b* increases because having more hash tables increases the probability of finding two fingerprints in the same bucket even if they have moderate Jaccard similarity. But this comes at the expense of increased memory requirements, search runtime, and false detections (*23*). Figure S10C modifies *v*, keeping *r* and *b* constant; the curve moves to the right with steeper slope as *v* increases, requiring higher Jaccard similarity between fingerprint pairs for a successful search and a sharper cutoff between detections and nondetections.

To detect similar earthquakes in continuous data, our many-to-many search application of FAST uses every fingerprint in the database as a search query so that we can find all other fingerprints in the database that are similar to each query fingerprint, with near-linear runtime complexity: *O*((*N*_fp_)^1+ρ^), where 0 < ρ < 1. For this data set, using the parameters in Table 1 and with the number of hash functions increased to *r* = 7, we estimated ρ = 0.36, given the similarity search runtime *t* as a function of continuous data duration *d* from Fig. 4B; we assume a power law scaling *t* = *Cd*^(1+ρ)^, where we solved for the factors *C* and ρ with a least-squares linear fit in log space: log *t* = log *C* + (1+ρ)log *d*. Because there is a linear relationship between *d* and *N*_fp_, *O*(*d*^1+ρ^) ~ *O*((*N*_fp_)^1+ρ^). This is faster and more scalable than the quadratic runtime of autocorrelation: *O*(*N*^2^), with *N* > *N*_fp_. The output of similarity search is a list of pairs of similar fingerprint indices, which we convert into times in the continuous data, with associated FAST similarity values. We can visualize this list of pairs as a sparse, symmetric *N*_fp_ × *N*_fp_ similarity matrix (fig. S8). This matrix is sparse because LSH avoids searching for dissimilar fingerprint pairs, which constitute most of the possible pairs.

## Supplementary Material

http://advances.sciencemag.org/cgi/content/full/1/11/e1501057/DC1

## SUPPLEMENTARY MATERIALS

Supplementary material for this article is available at http://advances.sciencemag.org/cgi/content/full/1/11/e1501057/DC1 Continuous data time gaps Detection on synthetic data Reference code: Autocorrelation Near-repeat exclusion of similar pairs Postprocessing and thresholding Fig. S1. Illustration of comparison between many-to-many search methods for similar pairs of seismic events. Fig. S2. Twenty-second catalog earthquake waveforms, ordered by event time in 1 week of continuous data from CCOB.EHN (bandpass, 4 to 10 Hz). Fig. S3. Catalog events missed by FAST, detected by autocorrelation. Fig. S4. Twenty-second new (uncataloged) earthquake waveforms detected by FAST, ordered by event time in 1 week of continuous data from CCOB.EHN (bandpass, 4 to 10 Hz); FAST found a total of 68 new events. Fig. S5. FAST detection errors. Fig. S6. Example of uncataloged earthquake detected by FAST, missed by autocorrelation. Fig. S7. Histogram of similar fingerprint pairs output from FAST. Fig. S8. Schematic illustration of FAST output as a similarity matrix for one channel of continuous seismic data. Fig. S9. CC and Jaccard similarity for two similar earthquakes. Fig. S10. Theoretical probability of a successful search as a function of Jaccard similarity. Fig. S11. Synthetic data generation. Fig. S12. Hypothetical precision-recall curves from three different algorithms. Fig. S13. Synthetic test results for three different scaling factors *c*: 0.05 (top), 0.03 (center), 0.01 (bottom), with snr values provided. Table S1. Autocorrelation input parameters. Table S2. NCSN catalog events. Table S3. Scaling test days. Table S4. Example of near-duplicate fingerprint pairs detected by FAST, which represent the same pair with slight time offsets. Reference (*44*)
