# Supplementary material for: Earthquake detection through computationally efficient similarity search
Source: Sci Adv. 2015 Dec 4;1(11):e1501057. doi: 10.1126/sciadv.1501057 (PMC4672764; doi:10.1126/sciadv.1501057)
Supplement: http://advances.sciencemag.org/cgi/content/full/1/11/e1501057/DC1 [file supp_1_11_e1501057__index.html]

Science Advances | Science Advances

## Supplementary Materials

**This PDF file includes:**

- Continuous data time gaps
- Detection on synthetic data
- Reference code: Autocorrelation
- Near-repeat exclusion of similar pairs
- Postprocessing and thresholding
- Fig. S1. Illustration of comparison between many-to-many search methods for similar pairs of seismic events.
- Fig. S2. Twenty-second catalog earthquake waveforms, ordered by event time in 1 week of continuous data from CCOB.EHN (bandpass, 4 to 10 Hz).
- Fig. S3. Catalog events missed by FAST, detected by autocorrelation.
- Fig. S4. Twenty-second new (uncataloged) earthquake waveforms detected by FAST, ordered by event time in 1 week of continuous data from CCOB.EHN (bandpass, 4 to 10 Hz); FAST found a total of 68 new events.
- Fig. S5. FAST detection errors.
- Fig. S6. Example of uncataloged earthquake detected by FAST, missed by autocorrelation.
- Fig. S7. Histogram of similar fingerprint pairs output from FAST.
- Fig. S8. Schematic illustration of FAST output as a similarity matrix for one channel of continuous seismic data.
- Fig. S9. CC and Jaccard similarity for two similar earthquakes.
- Fig. S10. Theoretical probability of a successful search as a function of Jaccard similarity.
- Fig. S11. Synthetic data generation.
- Fig. S12. Hypothetical precision-recall curves from three different algorithms.
- Fig. S13. Synthetic test results for three different scaling factors *c*: 0.05 (top), 0.03 (center), 0.01 (bottom), with snr values provided.
- Table S1. Autocorrelation input parameters.
- Table S2. NCSN catalog events.
- Table S3. Scaling test days.
- Table S4. Example of near-duplicate fingerprint pairs detected by FAST, which represent the same pair with slight time offsets.
- Reference (*44*)

Download PDF

**Files in this Data Supplement:**

- Adobe PDF - 1501057\_SM.pdf
