# Supplementary material for: Earthquake detection through computationally efficient similarity search
Source: Sci Adv. 2015 Dec 4;1(11):e1501057. doi: 10.1126/sciadv.1501057 (PMC4672764; doi:10.1126/sciadv.1501057)
Supplement: http://advances.sciencemag.org/cgi/content/full/1/11/e1501057/DC1 [file 1501057_SM.pdf]

Science Advances

AAAS

advances.sciencemag.org/cgi/content/full/1/11/e1501057/DC1

Supplementary Materials for

Earthquake detection through computationally efficient similarity search

Clara E. Yoon, Ossian O'Reilly, Karianne J. Bergen, Gregory C. Beroza

Published 4 December 2015, *Sci. Adv.* **1**, e1501057 (2015)
DOI: 10.1126/sciadv.1501057

The PDF file includes:

Continuous data time gaps
Detection on synthetic data
Reference code: Autocorrelation
Near-repeat exclusion of similar pairs
Postprocessing and thresholding
Fig. S1. Illustration of comparison between many-to-many search methods for similar pairs of seismic events.
Fig. S2. Twenty-second catalog earthquake waveforms, ordered by event time in 1 week of continuous data from CCOB.EHN (bandpass, 4 to 10 Hz).
Fig. S3. Catalog events missed by FAST, detected by autocorrelation.
Fig. S4. Twenty-second new (uncataloged) earthquake waveforms detected by FAST, ordered by event time in 1 week of continuous data from CCOB.EHN (bandpass, 4 to 10 Hz); FAST found a total of 68 new events.
Fig. S5. FAST detection errors.
Fig. S6. Example of uncataloged earthquake detected by FAST, missed by autocorrelation.
Fig. S7. Histogram of similar fingerprint pairs output from FAST.
Fig. S8. Schematic illustration of FAST output as a similarity matrix for one channel of continuous seismic data.
Fig. S9. CC and Jaccard similarity for two similar earthquakes.
Fig. S10. Theoretical probability of a successful search as a function of Jaccard similarity.
Fig. S11. Synthetic data generation.
Fig. S12. Hypothetical precision-recall curves from three different algorithms.
Fig. S13. Synthetic test results for three different scaling factors *c*: 0.05 (top), 0.03 (center), 0.01 (bottom), with snr values provided.

Table S1. Autocorrelation input parameters.
Table S2. NCSN catalog events.
Table S3. Scaling test days.
Table S4. Example of near-duplicate fingerprint pairs detected by FAST, which represent the same pair with slight time offsets.
Reference (*44*)

Supplementary Materials

Continuous Data Time Gaps

The selected week of continuous data from the NCEDC contained 7 time gaps, with the longest time gap around 14 minutes in duration. We stitched together the time series data, and placed uncorrelated white Gaussian noise in the time gaps, scaled by the mean and standard deviation of 1000 data samples on either end of the time gap. We confirmed that FAST did not detect any spurious events in or near the time gaps filled with synthetic noise.

Detection on Synthetic Data

We performed a synthetic test to compare objectively the detection performance of FAST against a reference autocorrelation code. Our synthetic data consisted of scaled-down earthquake waveforms inserted at known times into noisy seismic data (*7, 8*), which provides ground truth. We extracted broadband noise from the N-S component of CCOB (**Figure S11**A) during the first 12 hours of 2011-01-09. To simulate a repeating earthquake signal that FAST and autocorrelation should detect, we took a 10-second catalog earthquake waveform at 553-563 s (**Figure S11**B) in the CCOB data, multiplied it by a scaling factor c, and inserted it 24 different times into the noisy data. We also added a non-repeating earthquake signal, which we do not expect either algorithm to detect, by taking a different 10-second earthquake waveform (**Figure S11**C), scaling it by the same factor c, and planting it once into the noisy data.

We define the snr for the synthetic data as the ratio of the signal power to the noise power:

$$snr = \frac{P_{signal}}{P_{noise}} = \left(\frac{A_{signal}}{A_{noise}}\right)^2 \qquad (9)$$

where the signal amplitude A_{signal} and noise amplitude A_{noise} are root-mean-squared (rms) values

calculated from 10-second time windows (a_1, a_2, … , a_n), with n = 200 samples (for 20 sps data):

$$A_{rms} = \sqrt{\frac{1}{n}\left(a_1^2 + a_2^2 + \cdots + a_n^2\right)} \quad (10)$$

To compute A_{signal}, we used the 10-second catalog earthquake waveform at 553-563 s, scaled by the factor c. For A_{noise}, we computed the average of the A_{rms} values for 10-second time windows at the 24 times in the noisy data, prior to inserting the repeating earthquake signal.

Since we have ground truth for the synthetic data, we can quantify detection errors made by autocorrelation and FAST as we adjust their detection thresholds. At one particular threshold, classifications are correct when the algorithm detects an event where one truly was planted (true positive: TP), or fails to detect an event where one was not planted (true negative: TN). Classification errors occur when we falsely detect an event where one was not planted (false positive: FP), or we fail to detect an event where one actually was planted (false negative: FN).

Precision is the fraction of identified detections that are true event detections (*44*):

$$Precision = \frac{TP}{TP + FP} \quad (11)$$

and recall is the fraction of true events that were correctly identified as event detections (*44*):

$$Recall = \frac{TP}{TP + FN} \quad (12)$$

Changing the detection threshold results in different values for TP and FP, which can be used to trace out a curve called the precision-recall curve. **Figure S12** shows hypothetical precision-recall curves from three different algorithms. With perfect performance, where both FP and FN are zero, precision and recall are equal to one, at the upper right corner; however, there usually is a trade-off between these metrics, depending on the detection threshold. If the threshold is too low, recall will be high since we do not miss any events, but precision will be low since there

will be an increase in false detections. But if the threshold is too high, precision will be high since false detections are rejected, but recall may be low since we may also fail to detect actual events. We should set the threshold to the best compromise for the particular application.

Figure S13 displays synthetic test detection results, where the repeating earthquake signal was scaled by three different factors *c*: 0.05, 0.03, 0.01, with associated *snr* values from Eq. 9 and 10. We show synthetic data with planted, scaled waveforms and detection results as precision-recall curves for both autocorrelation and FAST. Table 1 contains FAST parameter values that we used, and **Table S1** displays parameters for the reference autocorrelation code. These are the same values used for detection in real data, except here we varied event detection thresholds to compute precision-recall curves; these thresholds are in units of CC for autocorrelation and FAST similarity for FAST. For $c = 0.05$ ($snr = 7.37$), both autocorrelation and FAST achieve perfect precision and recall (**Figure S13**B), finding all 24 planted events without any false positives. For $c = 0.03$ ($snr = 2.65$), autocorrelation still has perfect precision and recall, but FAST starts to trade off precision against recall (**Figure S13**D). For $c = 0.01$ ($snr = 0.29$), neither autocorrelation nor FAST detect any of the planted waveforms (**Figure S13**F). As expected, neither autocorrelation nor FAST detected the non-repeating planted waveform. We conclude from the synthetic test results that autocorrelation is a more sensitive detector than FAST for detecting low *snr* signals. But FAST performs on par with autocorrelation for moderate values of *snr*, with a significantly reduced computational cost. This is consistent with the fact that FAST makes approximations in both the feature extraction and similarity search steps, while autocorrelation performs a comprehensive, precise comparison.

Reference Code: Autocorrelation

Autocorrelation partitions the continuous data into *N* short overlapping time windows, and

computes $N(N\text{-}1)/2$ normalized CC values between all possible window pairs using Eq. 1. Using a time window length of M = 200 samples (10 s), and τ_w = 2 samples (0.1 s) as the lag between adjacent time windows (**Table S1**), we found that N = 6,047,901, which is about 10 times greater than N_{fp} = 604,781. This factor of 10 difference is attributed to the use of a 1 s lag between FAST fingerprints, compared to a 0.1 s lag for autocorrelation.

We use a "sliding window" implementation of autocorrelation that avoids redundant computation of the dot product in the CC (Eq. 1). The dot product can be broken up into three parts: two small boundary segments and one large interior segment, since there is a large amount of overlap between adjacent time windows. We compute the dot product for the next window pair by reusing the computation from the previous interior segment and adjusting for boundary segments. Our autocorrelation implementation is configured to run in parallel on up to 1000 processors with the Message Passing Interface (MPI), although we report runtime results on 1 processor for a fair comparison with FAST.

Our autocorrelation code outputs only time window pairs with CC above an initial threshold, defined by βd, where we used β = 5 and d is the median absolute deviation (MAD) defined with the L1 matrix norm as:

$$d = \frac{1}{N^2}\left\| \underline{CC} \right\|_1 = \frac{1}{N^2}\sum_{a=1}^{N}\sum_{b=1}^{N}\left| CC(\vec{a},\vec{b}) \right| \tag{13}$$

where $CC(\vec{a},\vec{b})$ is the CC between windows $\vec{a}$ and $\vec{b}$ in Eq. 1. We assume a normal distribution of correlation coefficients for our large value of N from the central limit theorem, but use MAD as a detection statistic because it is more robust to outliers (*8*).

Near-Repeat Exclusion of Similar Pairs

A query fingerprint will always identify itself as a similar fingerprint in the database, but this

match is trivial and of no interest to detection. Also, we are not interested in “near-repeat” pairs where a fingerprint is reported as similar to itself, but offset by a few time samples. Therefore, we use a “near-repeat exclusion” parameter, $n_r = 5$, to avoid returning any fingerprint within n_r samples of the search query fingerprint as a matching similar fingerprint. Since the fingerprint lag is 1 s, $n_r = 5$ samples is equivalent to 5 seconds (Table 1).

In the same way, we want to avoid autocorrelation output pairs that consist of a time window correlated with itself, so we use a “near-repeat exclusion” parameter, $n_r = 50$, to avoid returning any time window within n_r samples of the current window as a similar waveform. For a 0.1 s time window lag, $n_r = 50$ samples is equivalent to 5 seconds (**Table S1**).

Postprocessing and Thresholding

Post-processing and thresholding are required to convert a list of pairs of similar fingerprint times to a list of earthquake detection times. First, the list of pairs can have near-duplicate pairs with FAST similarity above the event detection threshold, when they really represent the same pair with slight time offsets (red squares in **Figure S8**). For example, **Table S4** shows a list of near-duplicate pairs that actually represent a single pair, so we keep only the single pair with the highest similarity 0.57, and remove the rest of the pairs within 21 seconds of the times for the highest similarity pair (Table 1).

After removing near-duplicate pairs, we create a list of event detection times. We sort the pairs in decreasing order of similarity, then add each event in the pair to the detection list. Sometimes we encounter a near-duplicate event: for example, pair (245266 s, 1335 s) has similarity 0.41, so we add both events to the detection list, then later we have pair (1332 s, 547 s), with lower similarity 0.39. We classify the 1332 s event as a near-duplicate of the 1335 s event, since they are within 21 seconds of each other (Table 1), and we do not add the 1332 s

event to the list. After removing near-duplicate events, we have a list of earthquakes detected by FAST; each event is defined by its time in the continuous data, and its FAST similarity.

We apply these same methods to convert a list of pairs of similar time windows, output from autocorrelation, to a list of detection times with CC values. We use a 21-second window to eliminate near-duplicate pairs and events (Table 1, **Table S1**): the minimum time required for a 10 s long fingerprint (Table 1) and a 10 s long autocorrelation window (**Table S1**) to avoid overlap.

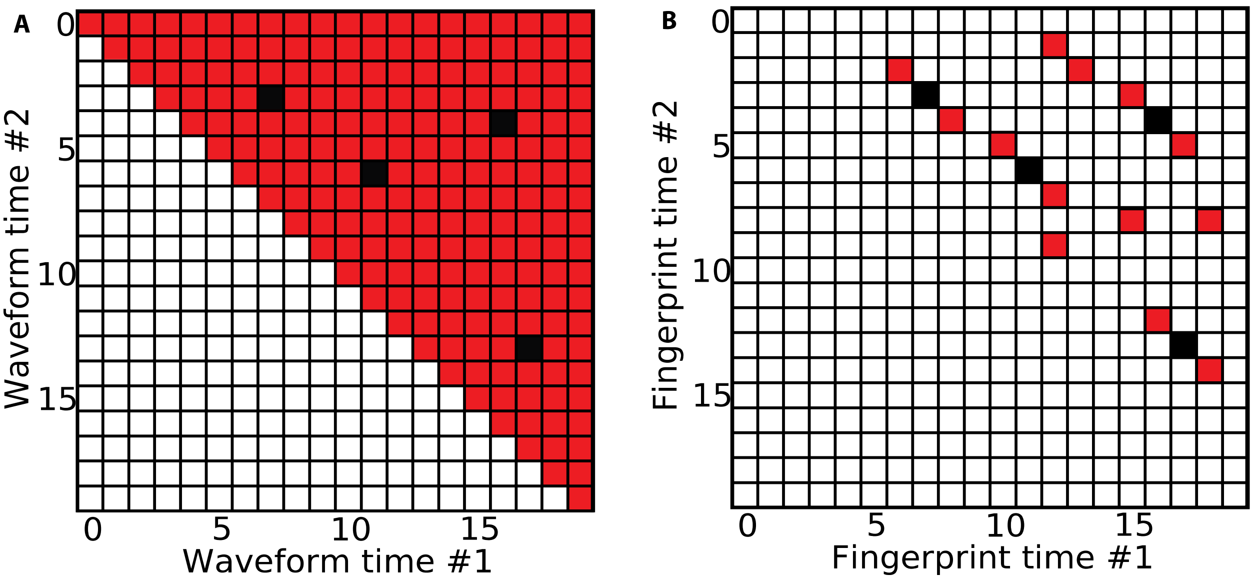

Figure S1: Illustration of comparison between many-to-many search methods for similar pairs of seismic events. Each grid square represents a pair of waveforms (or fingerprints) from two different times in the continuous data. Only upper triangular parts of these symmetric matrices are shown. **(A)** Autocorrelation requires comparing all possible pairs of waveforms (red, black) to find a tiny fraction of highly similar events (black). Most of the computational effort is wasted on dissimilar event pairs (red), where the number of pairs grows quadratically with the number of waveforms *N*. **(B)** FAST uses LSH to focus on a small number of candidate pairs (red, black) that are likely to be highly similar, in order to find seismic event pairs (black) while avoiding wasteful computation of dissimilar pairs (white).

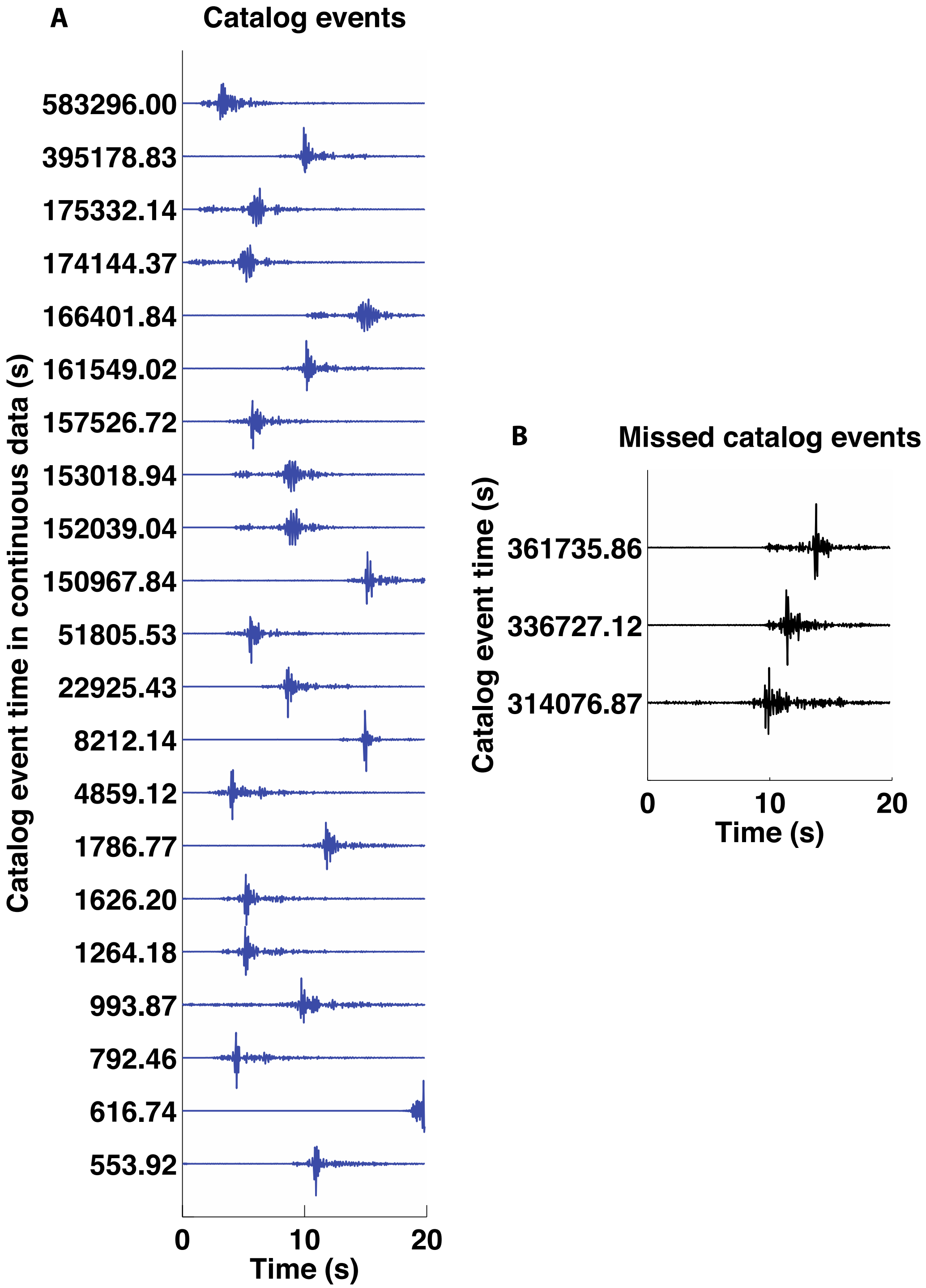

Figure S2: Twenty-second catalog earthquake waveforms, ordered by event time in 1 week of continuous data from CCOB.EHN (bandpass, 4 to 10 Hz). (A) FAST detected 21 (blue) out of 24 catalog events within the region of interest in Figure 2. **(B)** False negatives: FAST did not detect 3 (black) out of 24 catalog events in this data. Autocorrelation detected all 24 catalog events.

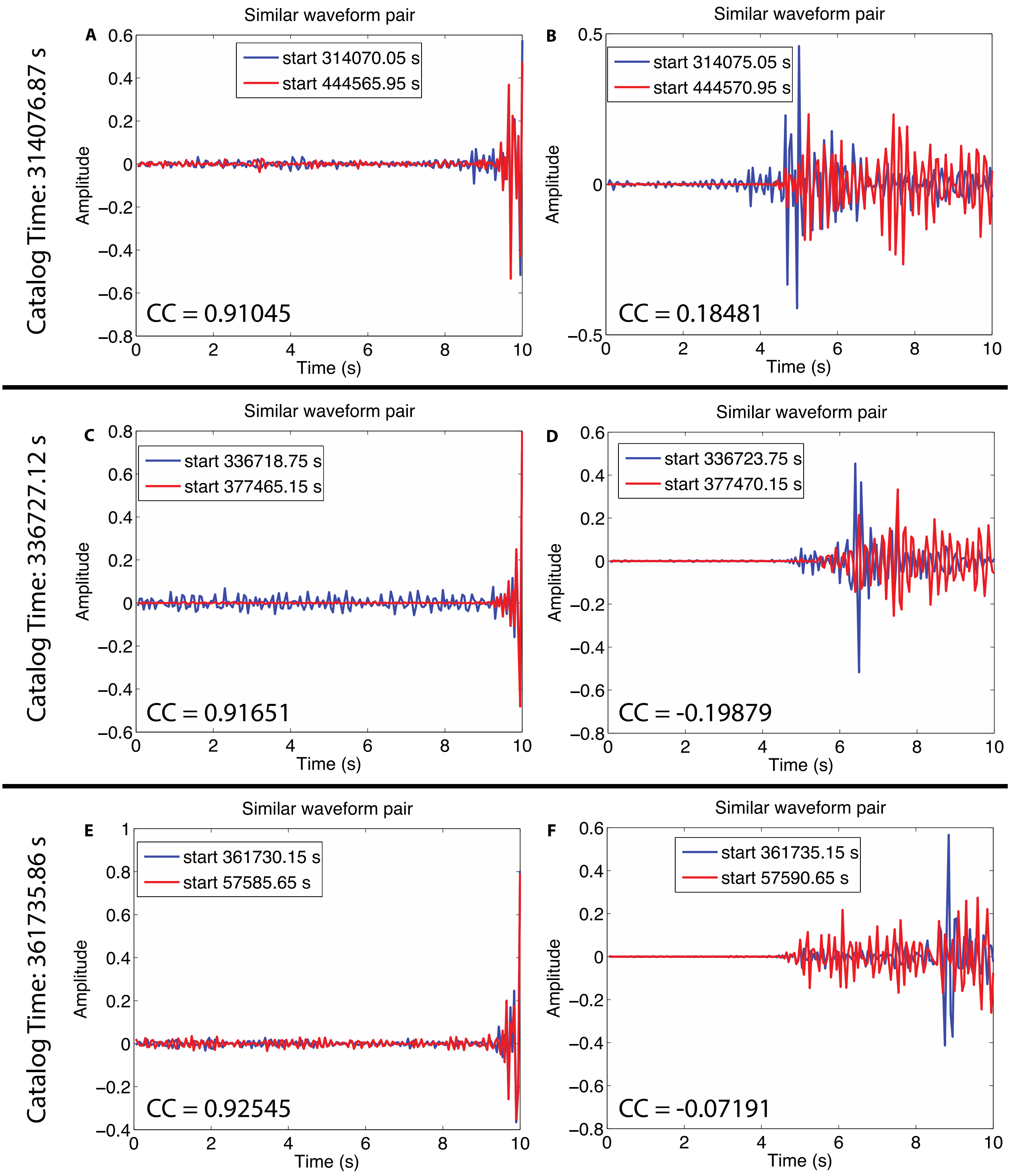

Figure S3: Catalog events missed by FAST, detected by autocorrelation. (Left column) Autocorrelation found these 3 catalog events (blue) because their initial phase arrival matched that of another earthquake (red) with high CC above 0.9. (Right column) Five seconds later, the normalized waveforms of the catalog event (blue) and its matching earthquake (red) are not

similar, with low CC values.

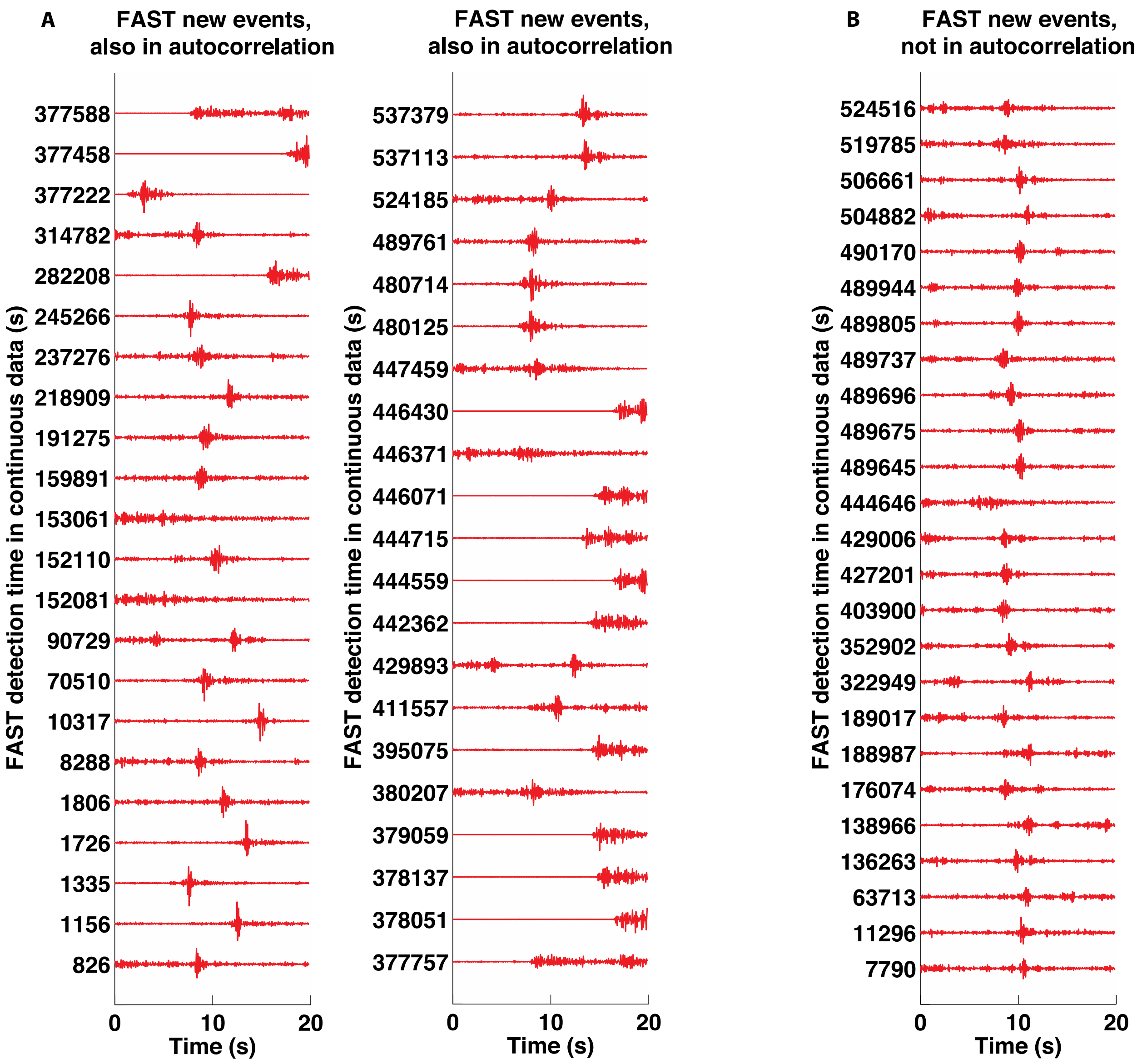

Figure S4: Twenty-second new (uncataloged) earthquake waveforms detected by FAST, ordered by event time in 1 week of continuous data from CCOB.EHN (bandpass, 4 to 10 Hz); FAST found a total of 68 new events. (A) FAST detected 43 new events that autocorrelation also found. **(B)** FAST detected 25 new events that were missed by autocorrelation.

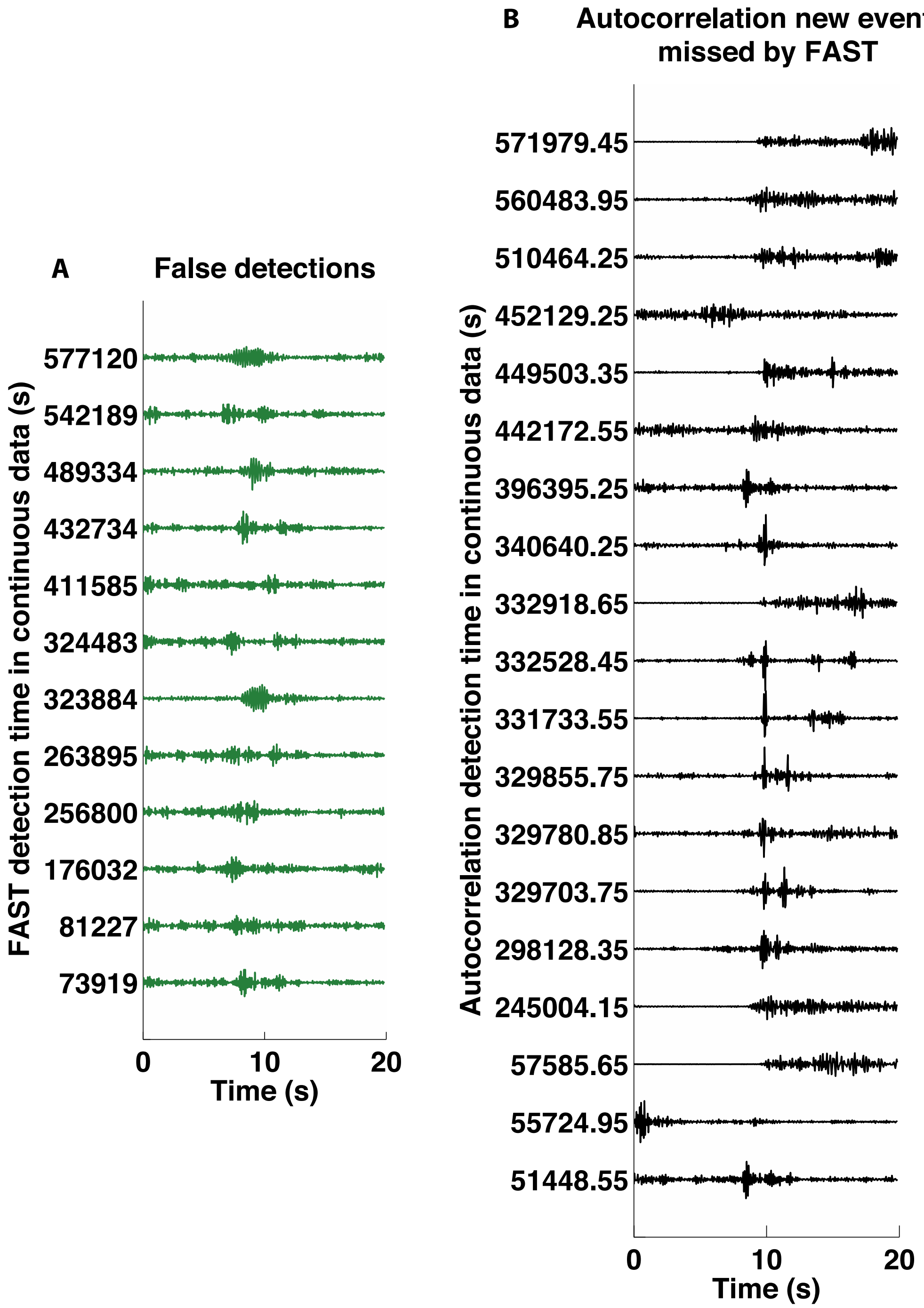

Figure S5: FAST detection errors. 20-second waveforms ordered by detection time in one week of continuous data from CCOB.EHN, bandpass 4-10 Hz. **(A)** False positives: FAST returned 12 detections (green), not found by autocorrelation, that we classified as false detections (noise) upon visual inspection. **(B)** False negatives: FAST missed 19 uncataloged events (black) found by autocorrelation, confirmed by visual inspection to be legitimate earthquakes, so these are missed detections.

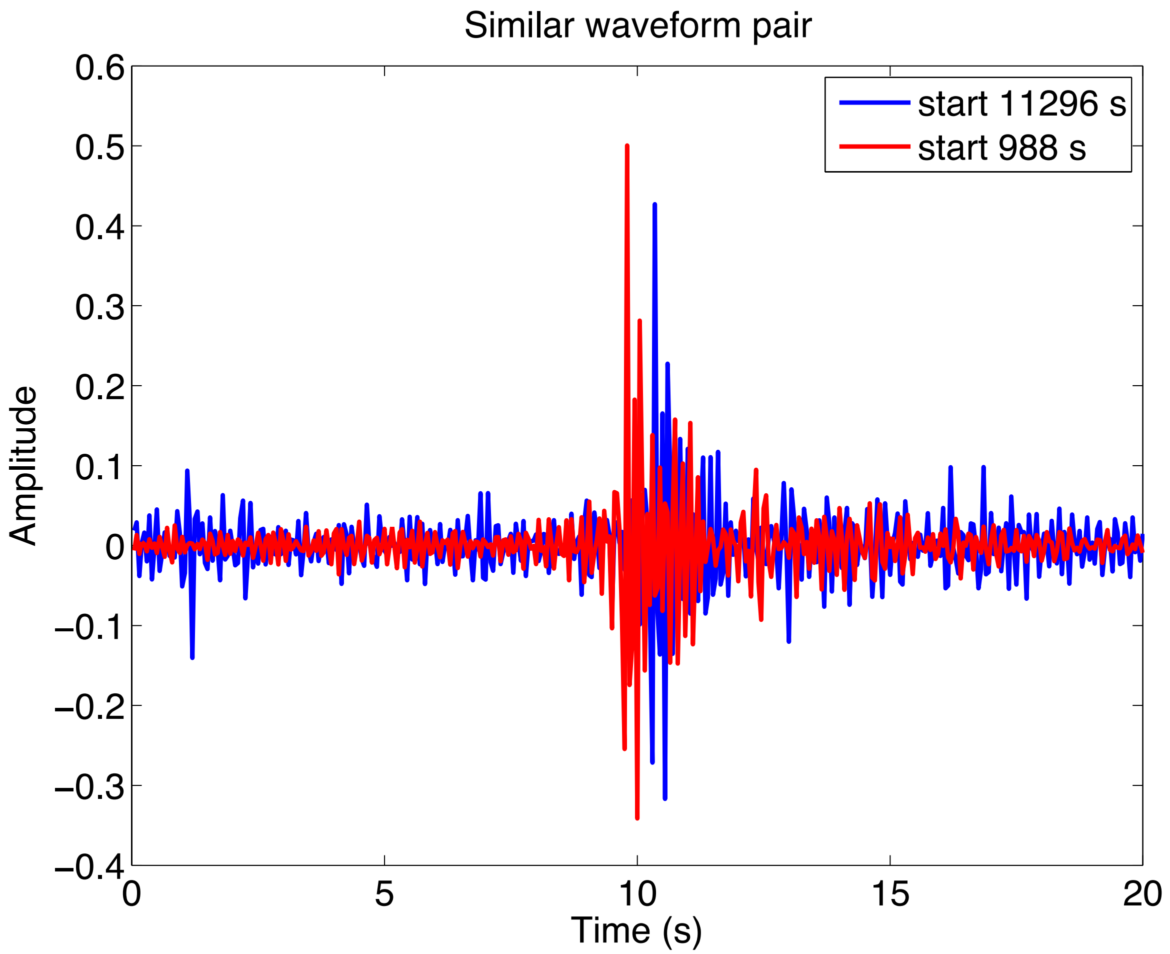

Figure S6: Example of uncataloged earthquake detected by FAST, missed by autocorrelation. New uncataloged event (11296 s, blue) was detected by FAST to be similar to a catalog event (988 s, red), but was not detected by autocorrelation. The overall waveform shapes are similar, but their alignment is not precise.

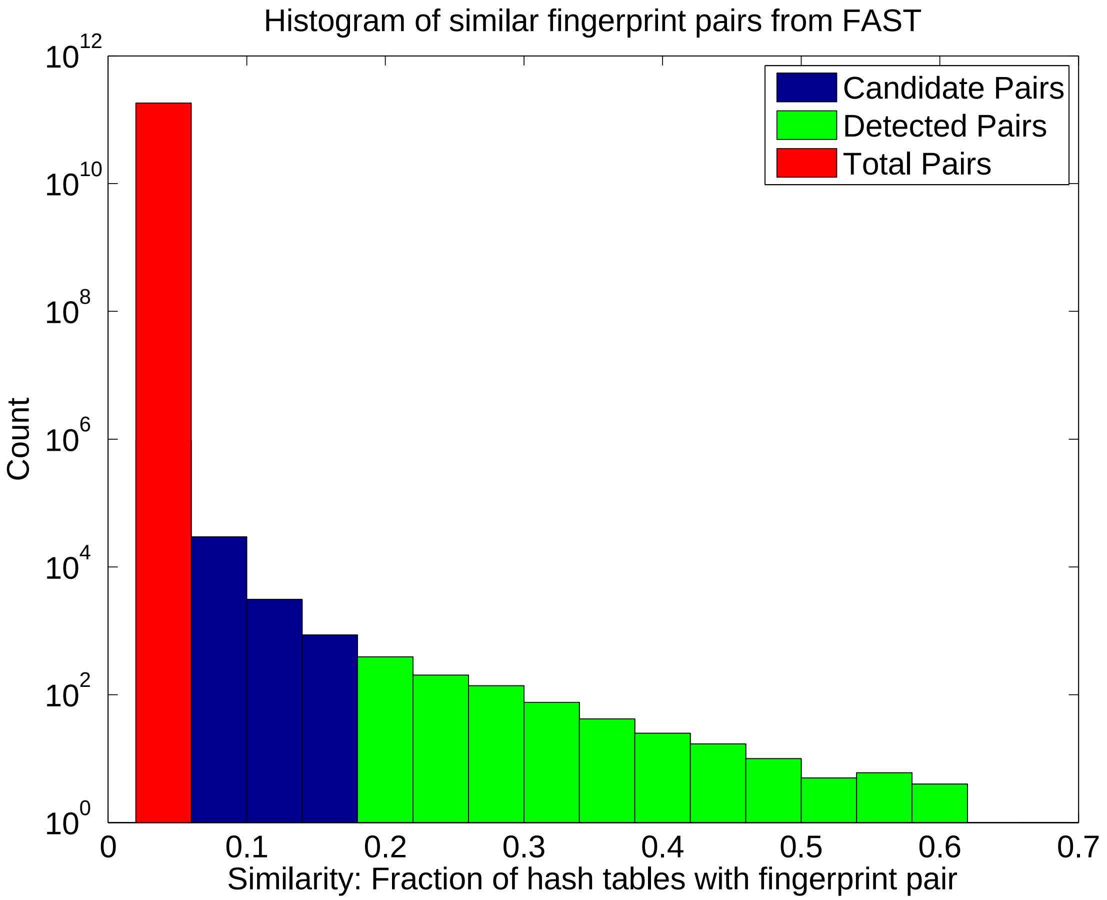

Figure S7: Histogram of similar fingerprint pairs output from FAST. It is binned by FAST similarity, with bin size 0.04. The number of pairs in each bin is on a log scale, and near-duplicate pairs are included. For N_{fp} = 604,781 fingerprints, there are $N_{fp}(N_{fp} - 1)/2 \sim 1.8 \cdot 10^{11}$ possible fingerprint pairs (red). FAST outputs 978,904 candidate pairs (blue, green) with similarity of at least the initial threshold of 0.04 (Table 1), which constitute only 0.0005% of the total number of possible pairs, although the database stores additional pairs in memory. After applying the higher event detection threshold of 0.19 (Table 1), 918 pairs remain (green). Further post-processing results in a list of 101 detections, consisting of 89 true events and 12 false detections.

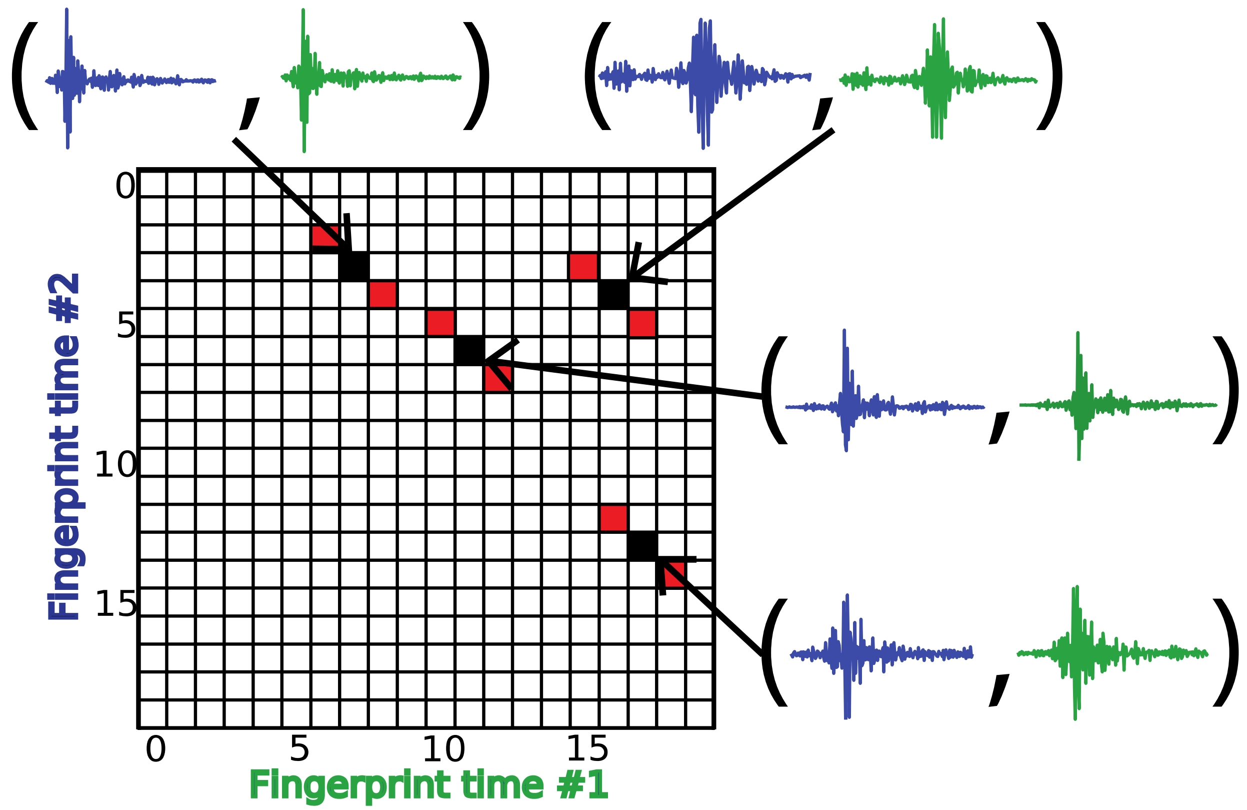

Figure S8: Schematic illustration of FAST output as a similarity matrix for one channel of continuous seismic data. As in Figure S1, each square represents a pair of fingerprints (which can be mapped back to waveforms) from two different times (blue, green) in the continuous data. This symmetric matrix is very sparse, since LSH restricts our search to highly similar fingerprint pairs; black squares with high similarity indicate when similar waveforms occur. The FAST similarity metric is the fraction of hash tables containing each fingerprint pair in the same hash bucket. Further processing and thresholding, including removal of near-duplicate pairs (red squares), is required to obtain a list of event detection times.

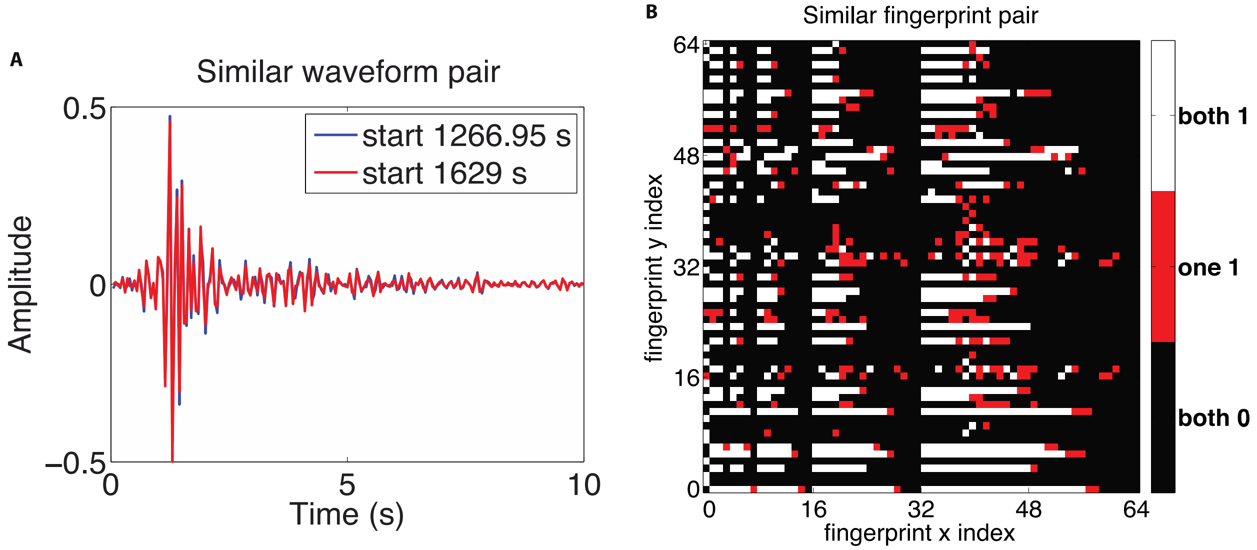

Figure S9: CC and Jaccard similarity for two similar earthquakes. (A) Two similar normalized earthquake waveforms, from 1266.95 s (blue) and 1629 s (red) in the CCOB.EHN continuous data on 2011-01-08, have a high CC = 0.9808 (Eq. 1), overlapping almost perfectly. **(B)** Corresponding fingerprints of these waveforms also have high overlap, with a high Jaccard similarity of 0.7544 (Eq. 5). White: both fingerprints are 1; red: one fingerprint is 1 and the other is 0; black: both fingerprints are 0. Here the Jaccard similarity J(A, B) equals the number of white elements (where both fingerprints A and B are 1) divided by the number of white elements plus the number of red elements (where either A or B is 1, but not both).

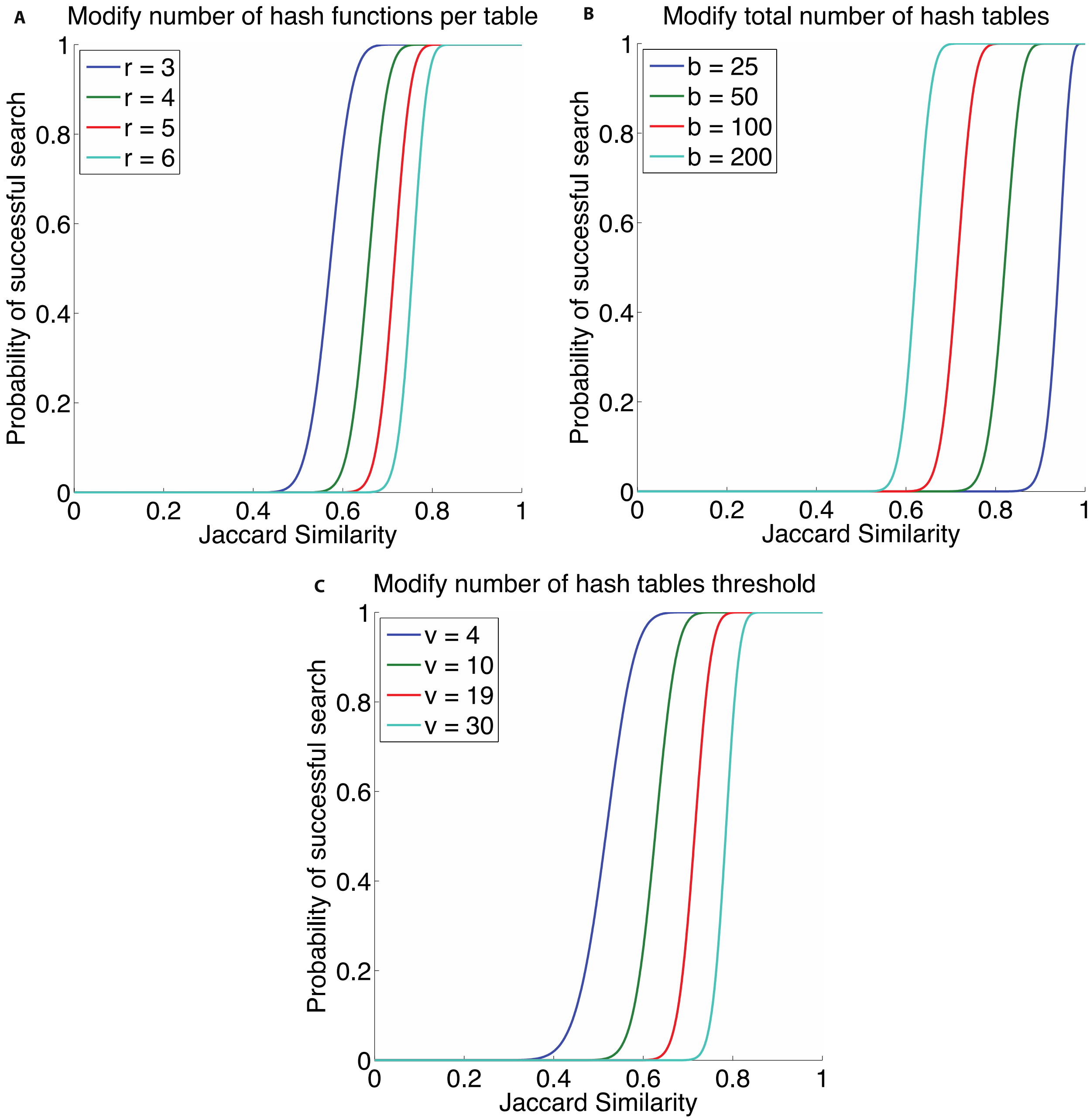

Figure S10: Theoretical probability of a successful search as a function of Jaccard similarity. We plot Eq. 7 while varying parameters r, b, v one at a time. Red curves (same in all plots) indicate our choice of FAST parameters (Table 1): $r = 5$, $b = 100$, and $v = 19$. **(A)** Modify number of hash functions per table r, keep $b = 100$ and $v = 19$ constant. As r increases, the curve shifts to the right, requiring higher Jaccard similarity for successful search. **(B)** Modify total number of hash tables b, keep $r = 5$ and $v = 19$ constant. As b increases, the curve moves to the left: more hash tables enable finding fingerprint pairs with lower Jaccard similarity. **(C)** Modify threshold for number of hash tables v with the pair in the same bucket, keep $r = 5$ and $b = 100$ constant. As v increases, the curve moves to the right, with steeper slope: Jaccard similarity must be higher for successful search, and there is a sharper cutoff for detections.

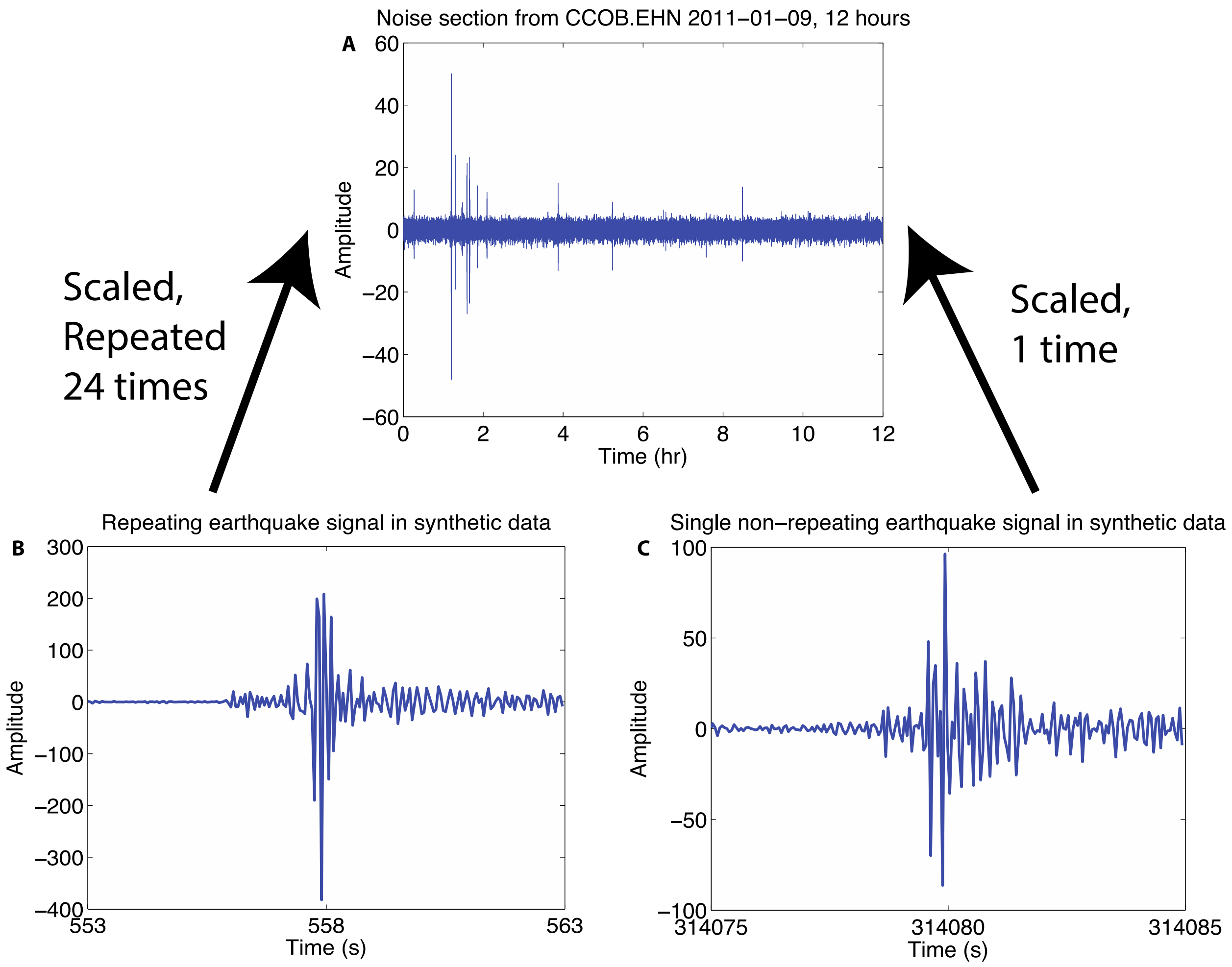

Figure S11: Synthetic data generation. (A) We extracted a noisy section of continuous data from the first 12 hours of 2011-01-09 at station CCOB.EHN - notice the low amplitude scale. **(B)** To simulate a repeating earthquake signal, we took a 10-second catalog earthquake waveform at 553-563 s, starting from 2011-01-08 00:00:00 in the CCOB.EHN data, multiplied it by a scaling factor *c*, and inserted it 24 times into the noisy data, every 30 minutes starting at 900 s: 900 s, 2700s, ... , 42300 s. **(C)** We also added a non-repeating earthquake signal by taking a 10-second earthquake waveform at 314075-314085 s, scaling it by the same factor *c*, and planting it once into the noisy data at 19800 s.

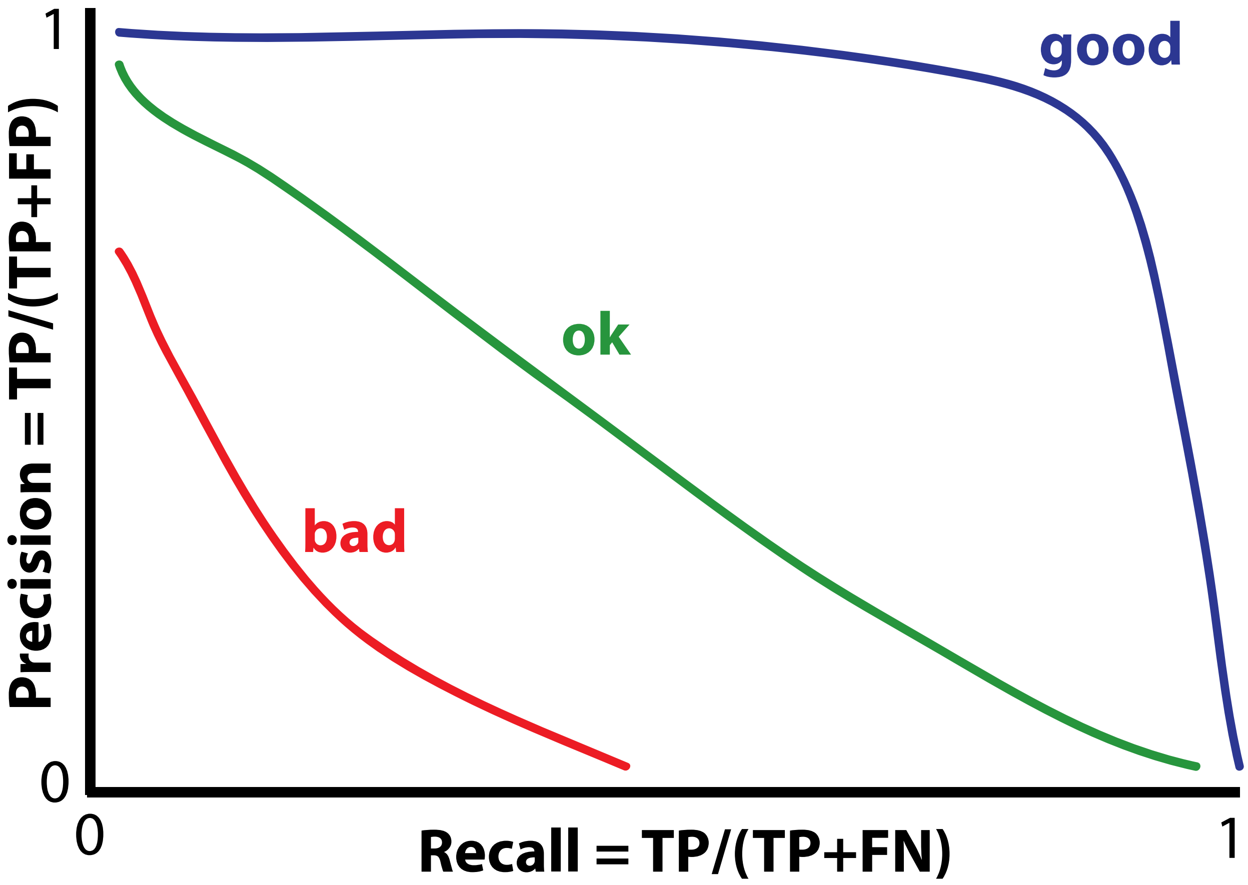

Figure S12: Hypothetical precision-recall curves from three different algorithms. Each point along the curve represents a different detection threshold. Ideally, if there are no FP or FN errors, both precision and recall would equal 1, so the curve would touch the upper-right corner. But there usually is a trade-off between these metrics depending on the detection threshold. The algorithm for the blue curve has the best detection performance since we can set a threshold such that both precision and recall are close to 1. The algorithm for the green curve is not as good: we can have high precision or high recall, but not both at the same time. The red curve displays the worst performance: both precision and recall are low, regardless of threshold.

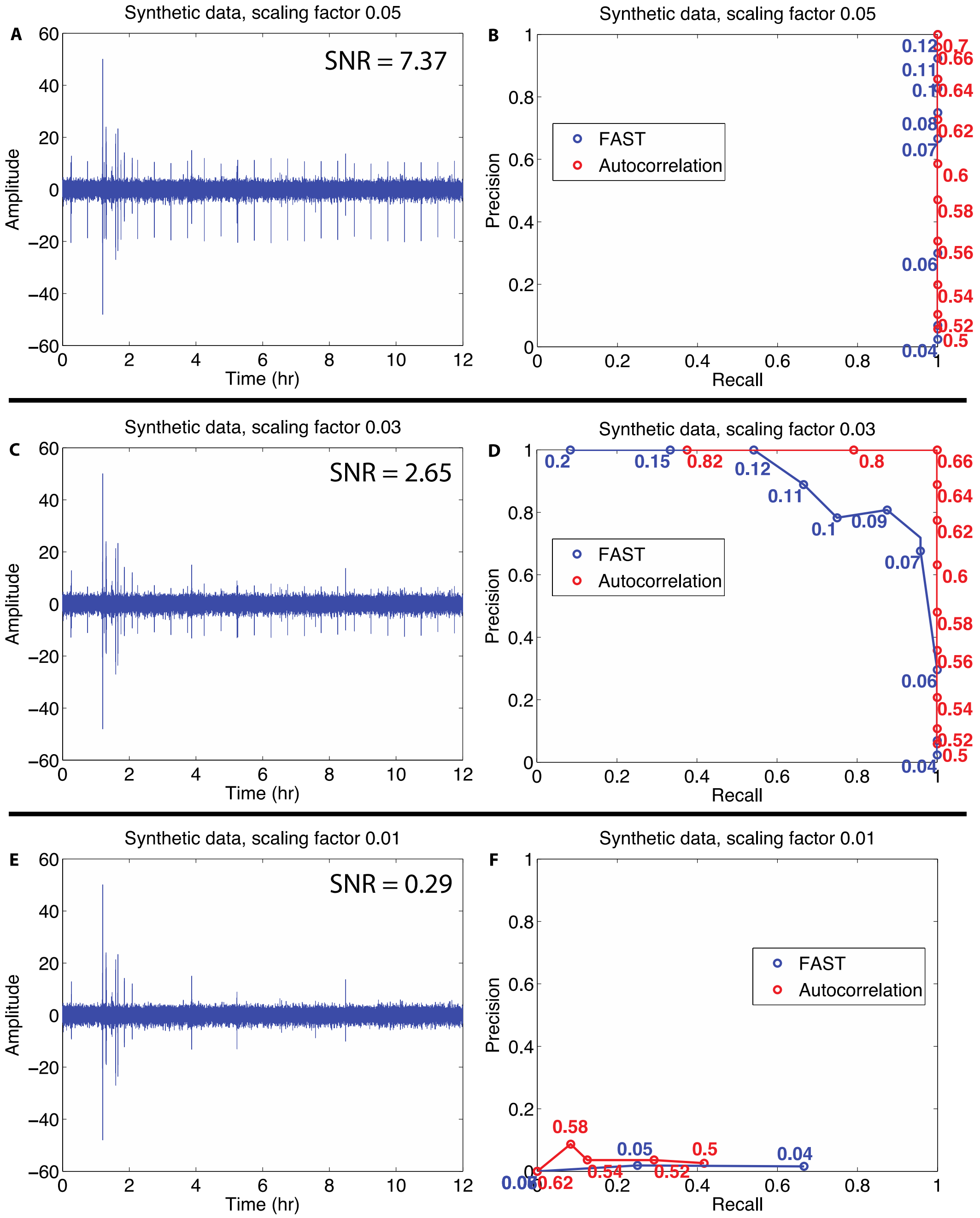

Figure S13: Synthetic test results for three different scaling factors *c*: 0.05 (top), 0.03 (center), 0.01 (bottom), with snr values provided. We show synthetic data with planted, scaled waveforms (left) and detection results as precision-recall curves (right) for both autocorrelation (red) and FAST (blue), generated by setting detection thresholds in terms of CC and FAST similarity, respectively. **(A)** For c = 0.05, planted waveforms are visible at every 30 minutes in the synthetic data; **(B)** both autocorrelation and FAST achieve perfect precision and recall, finding all 24 planted events without any false positives. **(C)** For c = 0.03, it is more difficult to spot the planted waveforms; **(D)** here autocorrelation still has perfect precision and recall, but FAST trades off precision against recall. **(E)** For c = 0.01, planted waveforms are below the noise level, and **(F)** both autocorrelation and FAST have poor detection performance.

Table S1: Autocorrelation input parameters. These were used for detection in synthetic data (except the event detection CC threshold), and in one week of CCOB.EHN data.

**Autocorrelation Parameter**	**Value**
Time series window length	200 samples (10 s)
Time series window lag	2 samples (0.1 s)
Similarity search: near-repeat exclusion parameter	50 samples (5 s)
Scale factor β for MAD, for initial threshold	5
Event detection CC threshold	0.818
Near-duplicate pair and event elimination time window	21 s
FAST and catalog comparison time window	19 s

Table S2: NCSN catalog events. Double-difference catalog events between 2011-01-08 00:00:00 and 2011-01-15 00:00:00, in region of interest between 37.1°-37.4° N, and 121.8°-121.5° W. "Catalog time" is time in seconds since 2011/01/08 00:00:00.

**Date**	**Time**	**Catalog Time (s)**	**Latitude**	**Longitude**	**Depth (km)**	**Magnitude**	**Magt**	**Event ID**
2011/01/08	00:09:13.92	553.92	37.28722	-121.66277	6.318	1.17	Md	71506870
2011/01/08	00:10:16.74	616.74	37.28678	-121.66014	6.212	4.10	Mw	71506865
2011/01/08	00:13:12.46	792.46	37.28462	-121.66060	6.326	2.53	Md	71506875
2011/01/08	00:16:33.87	993.87	37.28108	-121.65750	6.367	0.84	Md	71506885
2011/01/08	00:21:04.18	1264.18	37.28916	-121.66484	6.251	2.04	Md	71506890
2011/01/08	00:27:06.20	1626.20	37.28932	-121.66433	6.491	0.89	Md	71506895
2011/01/08	00:29:46.77	1786.77	37.29155	-121.66685	6.212	1.12	Md	71506900
2011/01/08	01:20:59.12	4859.12	37.28389	-121.66020	6.272	2.09	Md	71506910
2011/01/08	02:16:52.14	8212.14	37.24642	-121.63261	5.026	1.22	Md	71506925
2011/01/08	06:22:05.43	22925.43	37.29919	-121.67361	6.342	1.46	Md	71506995
2011/01/08	14:23:25.53	51805.53	37.29459	-121.66943	6.230	0.97	Md	71507125
2011/01/09	17:56:07.84	150967.84	37.26086	-121.64450	4.372	0.93	Md	71507625
2011/01/09	18:13:59.04	152039.04	37.12304	-121.52761	7.144	3.06	ML	71507630
2011/01/09	18:30:18.94	153018.94	37.12271	-121.52776	7.071	2.96	ML	71507640
2011/01/09	19:45:26.72	157526.72	37.29417	-121.66914	6.250	0.85	Md	71507675
2011/01/09	20:52:29.02	161549.02	37.29811	-121.67268	6.342	1.20	Md	71507705
2011/01/09	22:13:21.84	166401.84	37.12062	-121.52636	7.079	1.47	Md	71507720
2011/01/10	00:22:24.37	174144.37	37.12536	-121.52899	7.123	1.86	Md	71507750
2011/01/10	00:42:12.14	175332.14	37.12490	-121.52881	7.086	1.66	Md	71507765
2011/01/11	15:14:36.87	314076.87	37.27168	-121.65401	3.504	0.89	Md	71508480
2011/01/11	21:32:07.12	336727.12	37.28041	-121.66122	3.535	0.92	Md	71508655
2011/01/12	04:28:55.86	361735.86	37.13208	-121.57879	5.070	1.47	Md	71508765
2011/01/12	13:46:18.83	395178.83	37.29899	-121.67331	6.358	1.06	Md	71509005
2011/01/14	18:01:36.00	583296.00	37.29475	-121.67558	2.701	2.02	Md	71510000

Table S3: Scaling test days. Specific days of continuous data from CCOB.EHN used for the memory and runtime scaling tests in Figure 4.

**Data Duration**	**Specific days (inclusive) of continuous data**
1 day	2011-01-08
3 days	2011-01-08 to 2011-01-10
1 week (7 days)	2011-01-08 to 2011-01-14
2 weeks (14 days)	2011-01-08 to 2011-01-21
1 month (31 days)	2011-01-08 to 2011-02-07
3 months (90 days)	2011-01-01 to 2011-03-31
6 months (181 days)	2011-01-01 to 2011-06-30

Table S4: Example of near-duplicate fingerprint pairs detected by FAST, which represent the same pair with slight time offsets. We keep only the single pair (395173, 161543) with the highest similarity 0.57, and discard the rest.

**Time 1 (s)**	**Time 2 (s)**	**Similarity**
395170	161540	0.49
395171	161541	0.51
395172	161542	0.5
395173	161543	0.57
395174	161544	0.4
395175	161545	0.42
